# Enhancing Therapeutic Efficacy of Double Negative T Cells against Acute Myeloid Leukemia Using Idelalisib

**DOI:** 10.3390/cancers13205039

**Published:** 2021-10-09

**Authors:** Hyeonjeong Kang, Jong Bok Lee, Ismat Khatri, Yoosu Na, Cheryl D’Souza, Andrea Arruda, Mark D. Minden, Li Zhang

**Affiliations:** 1 Toronto General Hospital Research Institute, University Health Network, Toronto, ON M5G 2C4, Canada; heidi.kang@mail.utoronto.ca (H.K.); jongbok.lee@uhnresearch.ca (J.B.L.); Ismat.khatri@uhnresearch.ca (I.K.); Yoosu.na@uhnresearch.ca (Y.N.); Cheryl.Dsouza@uhnresearch.ca (C.D.); 2 Department of Laboratory Medicine and Pathobiology, University of Toronto, Toronto, ON M5S 1A1, Canada; 3 Princess Margaret Cancer Centre, University Health Network, Toronto, ON M5G 2C4, Canada; Andrea.arruda@uhnresearch.ca (A.A.); mark.minden@uhn.ca (M.D.M.); 4 Department of Immunology, University of Toronto, Toronto, ON M5S 1A1, Canada

**Keywords:** double negative T cell, adoptive T cell therapy, PI3K delta inhibitor, acute myeloid leukemia

## Abstract

**Simple Summary:**

Persistence of infused cells is an important factor that dictates the outcome of adoptive cellular therapy (ACT). DNT therapy is a novel form of ACT with promising result in treating relapsed or refractory AML in preclinical and early clinical studies. However, in vivo kinetics of human DNTs in cancer-bearing host have not been previously investigated. This study was the first to investigate the persistence of DNTs and ways to improve it in patient-derived xenograft models. DNTs persistence was observed up to 50 days in various organs of leukemia-bearing hosts. However, the detected DNT level was low while significant level of persisting AMLs was observed. To improve the in vivo persistence and therapeutic efficacy of DNTs, we expanded DNTs in the presence of an PI3Kδ inhibitor, idelalisib (Ide). Ide treatment of healthy donor-derived DNTs promoted early memory subsets and improved overall fitness, reducing exhaustion while improving viability. These Ide-induced attributes led to prolonged persistence of DNTs, resulting in superior anti-leukemic activity in vivo. Further, Ide-treated DNTs improved the durability of the treatment response. Collectively, our study highlights the importance of DNT persistence and Ide-mediated improvements in the overall fitness of DNTs, which promote longer persistence in vivo and better treatment outcome.

**Abstract:**

The double negative T cell (DNT) is a unique subset of T cells with potent anti-leukemic potential. Previously, DNT therapy has been shown to effectively target AML cells in patient-derived xenograft (PDX) models. Further, a recently completed phase I/IIa clinical study demonstrated the safety, feasibility, and potential efficacy in AML patients that relapsed after allogeneic hematopoietic stem cell transplantation. However, the persistence and durability of DNT-mediated anti-leukemic response is less well understood. In this study, we characterized the in vivo persistence of DNTs in PDX models. Further, we improved the efficacy and durability of DNT-mediated activity with phosphoinositide 3-kinase delta (PI3Kδ) inhibition. Mechanistically, DNTs treated with the PI3Kδ inhibitor, Idelalisib (Ide), exhibited early memory phenotype with superior viability and proliferative capacity but less cell exhaustion. Collectively, the findings from this study support the use of Ide-treated DNTs to improve its therapeutic outcome.

## 1. Introduction

AML is the most common form of adult acute leukemia with a poor overall 5-year survival rate of 30–45% for patients 60 years old or younger and 10–15% for elderly patients over 60 years of age [1,2]. Although induction chemotherapy is effective, high relapse rate and refractory disease contribute to the poor patient outcome. Currently, allogeneic hematopoietic stem cell transplantation (allo-HSCT) is the only form of treatment with sustained curative potential for chemotherapy-resistant AML patients, where the treatment effect is largely driven by the graft-versus-leukemia (GvL) activity of donor-derived immune cells [1]. However, severe transplant-associated toxicities, such as graft-versus-host disease (GvHD), hinder its clinical benefit for patients [3].

Utilizing the potency of GvL effect, immune cell-based therapy has been an active area of research to provide novel treatment options for high-risk AML patients. Of those, adoptive cellular therapy (ACT) using chimeric antigen receptor (CAR)-T cells and NK cells has demonstrated promising outcomes in early phase clinical trials [4,5,6,7,8,9]. However, only small cohorts of patients were treated with CAR-T cells, and, while effective, severe treatment-associated toxicities were observed [4,5,6,7]. In contrast, NK cell therapy was shown to be safe in multiple trials, but its clinical benefit was variable and largely confined as a consolidation therapy to prevent disease relapse, rather than to treat relapsed or refractory patients [1,8,9]. Additionally, high treatment-associated costs and difficulties in large scale manufacturing as well as poor persistence are some of the known logistical challenges of CAR-T and NK therapies, respectively [1,7].

Double negative T cells (DNTs) are a rare subset of mature peripheral T lymphocytes that expresses CD3 in the absence CD4 and CD8 expression. Murine DNTs have been shown to rescue mice from lethal doses of leukemia, and a correlative study showed that allo-HSCT patients with better DNT recovery have a lower risk of disease relapse [10,11]. To that end, our group developed methods to expand DNTs ex vivo and demonstrated that both patient- and donor-derived DNTs not only induce anti-leukemic activity against AML, but also target array of solid and hematological cancers by reducing tumour burden in various xenograft models without causing off tumour toxicities [12,13,14,15]. Interestingly, ex vivo expanded human DNTs exhibit an innate immune cell-like behaviour, where DNTs recognize cancer cells in an antigen non-specific manner using receptors such as NKG2D, DNAM-1, and TRAIL [13,14,16]. Importantly, we demonstrated that donor-derived DNTs fulfill the requirements of an off-the-shelf allogeneic T cell therapy without any genetic modifications. In particular, DNTs lack off-tumor activity, such as GvHD, and are resistant to host-versus-graft rejection [13,17]. Recently, a phase I/IIa clinical trial using allogeneic DNTs to treat AML patients who relapsed after allo-HSCT was completed (ChiCTR-IPR-1900022795). While these patients had extremely poor prognoses, DNT treatment achieved a 63.6% response rate with a 1-year overall survival of 40.6%. Further, DNTs did not induce any severe treatment-associated toxicity [18]. While these findings make DNT therapy an attractive form of ACT, the persistence and durability of DNT-mediated responses are less well characterized.

The advantage of cell-based therapy as a living drug stems from the persistence of infused cells, which mediate the treatment effect for a prolonged duration. Numerous studies consistently demonstrate the importance of infused cell persistence for favorable outcomes [19,20,21,22,23,24,25]. Recently, we demonstrated that ex vivo expanded human DNTs can persist in naïve immune-deficient mice for at least 14–21 days [17,26]. However, studies with a longer follow up have not been conducted. Further, the persistence of DNTs in leukemia-bearing hosts may differ from naïve mice, as leukemic blasts may provide stimulatory or inhibitory signals that affect the persistence of DNTs [27]. In this study, we first characterized the persistence of ex vivo expanded DNTs in leukemia-bearing mice. We also investigated whether Idelalisib (Ide), an FDA-approved phosphoinositide 3-kinase delta (PI3Kδ) inhibitor for lymphoma treatment, can enhance persistence of DNTs and provide more durable leukemia control, as well as the mechanisms involved.

## 2. Materials and Methods

### 2.1. Human Samples and Study Approval

Human blood from healthy donors was collected under the University Health Network (UHN), Toronto, ON, Canada, with Research Ethics Board (REB) approval (05-0221-T, latest approval on 12 May 2021). Frozen primary AML samples were obtained from the Leukemia Tissue Bank at Princess Margaret Cancer Centre (Toronto, ON, Canada) under UHN REB (01-0573, latest approval on 25 March 2021). All specimens were de-identified and given unique IDs before use. Patient information is provided in Supplemental Table S1. Animal studies were performed under approval by the Institutional Animal Care Committee of UHN (AUP: 741, latest approval on 4 June 2021) in accordance with the Canadian Council on Animal Care Guidelines.

### 2.2. Ex Vivo Expansion of DNTs and Ide Treatment

DNT was obtained and expanded as previously reported [16] in the presence or absence of Ide (Selleckchem, Houston, TX, USA). Briefly, blood from healthy donor was collected in sodium heparin-containing vacutainers (Becton, Dickinson and Company, Franklin Lakes, NJ, USA). Then, CD4^+^ and CD8^+^ T cells were depleted from healthy donor-derived PBMCs by incubating whole blood with RosetteSep human CD4 and CD8 depletion cocktail (StemCell Technologies, Vancouver, BC, Canada) followed by density gradient centrifugation to collect CD4- and CD8-depleted buffy coats. This method successfully isolates DNTs with >85% purity, and DNTs were cultured on anti-CD3 antibody-coated plates (OKT3-Biolegend, San Diego, CA, USA) in RPMI (ThermoFisher Scientific, Waltham, MA, USA) in the presence of 10% fetal bovine serum (Gibco, Waltham, MA, USA), 250 IU/mL of IL-2 (Proleukin-Novartis Pharmaceuticals, Basel, Switzerland), and with 1 μM of Ide or equivalent amount of DMSO for 3 days. Soluble OKT3, IL-2, and 1 μM of Ide (Ide-DNT) or equivalent amount of DMSO (ctrl-DNT) were added to the culture every 3–4 days. DNTs were used for all functional assays between day 14 and day 20 of expansion after removal of Ide by removing the supernatant after centrifugation and washing one more time with PBS.

### 2.3. Antibodies and Flow Cytometry

The following antibodies were used in this study: CD4-FITC or -APCcy7, CD56-PE, CD3-PEcy7 or -Alexa Flour700, CD8-APC or -APCcy7, CD45RA-FITC, CD95-APC, CD62L-PEcy7, CTLA4-PE, LAG3-PEcy7, TIM3-APC, CD45-FITC or -APCcy7, CD34-PE, CD33-APC, AnnexinV-Pacific Blue or -FITC, 7AAD, IFNy-PE, TNFα-APC, and DAPI. All listed anti-human antibodies were purchased from BioLegend. Data were obtained using Attune NxT flow Cytometer (ThermoFisher) and analyzed with Flowjo software (Tree Star, Inc., Ashland, OR, USA).

### 2.4. Patient-Derived Xenograft Models

Subsequently, 6–12 weeks old NOD.Cg-Prkdcscid Il2rgtm1Wjl/SzJ (NSG) mice were purchased from Jackson Laboratories (Bar Harbor, ME, USA) and housed in the Animal Resource Centre at UHN. Mice were sublethally irradiated (2.5 Gy) one day prior to intravenous injection of 2–3 × 10^6^ AML blasts obtained from patients. On day 17 and day 20, mice were injected with PBS or 20 × 10^6^ Ide-DNT or ctrl-DNTs with 10,000 IU of IL-2 intravenously and followed by weekly 10,000 IU of IL-2 intraperitoneal injections. Mice body weight was measured twice a week. On day 24–100, mice were euthanized and the bone marrow (BM), spleen, liver, and lung were harvested. For the liver and lung, small tissue sections were fixed in 10% formalin and sent for hematoxylin and eosin (H&E) staining. The rest of tissues were digested with collagenase D at 37 ℃ for 30 min, filtered through 40 µm cell strainer, and underwent density gradient centrifugation at 1200× *g* for 20 min. The buffy coats were collected to check for the frequency and number of DNTs and AML using flow cytometry, as described previously. To longitudinally monitor the DNT level in mice periphery, 75–100 µL of blood was collected from lateral saphenous vein weekly, and red blood cells were lysed using ammonium chloride (StemCell Technologies). Subsequently, DNT and AML levels were assessed by flow cytometry.

### 2.5. Cytokine Production Assay

Day 14–20 ex vivo expanded DNTs were cultured in medium for 4 h with anti-CD3/anti-CD28 dynabeads (Gibco, Waltham, MA, USA) and ×1 protein transport inhibitor cocktail (eBioscience, San Diego, CA, USA) following the manufacturer’s instructions. Cells were harvested after stimulation, fixed, and permeabilized with fixation buffer (BioLegend) and perm wash buffer (BioLegend), respectively. Fixed and permeabilized cells were intracellularly stained using anti-human cytokine antibodies as listed above.

### 2.6. In Vitro Cytotoxicity Assay

DNTs were co-cultured with AML cells at 0.25:1 to 4:1 effector-to-target ratios for 2 h or 20 h in 96-well plates and apoptosis of targeted cells was measured using flow cytometry. Specific killing of targets was calculated by

$$\frac{\%\mathrm{AnnexinV}\;\mathrm{of}\;\mathrm{AML}\;\mathrm{with}\;\mathrm{DNT}-\%\mathrm{AnnexinV}\;\mathrm{of}\;\mathrm{AML}\;\mathrm{without}\;\mathrm{DNT}}{100-\%\mathrm{AnnexinV}\;\mathrm{of}\;\mathrm{AML}\;\mathrm{without}\;\mathrm{DNT}}$$

and viability of effector cells was calculated by 100% − AnnexinV^+^ in cultures of DNTs with AML.

### 2.7. Real-Time PCR

RNA was isolated from day 14–20 ex vivo expanded DNTs using RNeasy Mini Kits (Qiagen, Hilden, Germany) following the manufacturer’s protocol. cDNA was synthesized from the isolated mRNA using QuantiTect Reverse Transcription Kit (Qiagen) according to the manufacturer’s protocol. Real-time PCR was performed on the LightCycler 480 Instrument (Roche Life Science, Penzberg, Germany) using the LightCycler 480 SYBR Green I Master (Roche). Data were analyzed using LightCycler 480 Software version 1.5. Primers that were used for all qPCR were obtained from Integrated DNA Technologies (Coralville, IA, USA) and are listed in Supplementary Table S2.

### 2.8. Statistical Analysis

All statistical analyses were performed using GraphPad Prism 5. The data were analyzed by two-tailed student’s *t* test, linear regression tests, and two-way ANOVA, where statistical significance was set as * *p* < 0.05, ** *p* < 0.01 and *** *p* < 0.001. Graphs are presented with mean ± SEM.

## 3. Results

### 3.1. DNT Persistence and Anti-Leukemic Activity in Leukemia-Bearing Hosts

A number of studies highlight the importance of infused cell persistence for a favourable clinical outcome of ACT [19,20,21,22,23,24,25]. Previously, we and others have demonstrated that adoptively transferred human DNTs could persist in naïve NSG mice for 2–3 weeks [17,26]. However, DNT engraftment in a leukemia-bearing host may differ from those seen in naïve mice, as AML can hamper immune cell survival and anti-leukemic activity via immunosuppressive function or promote their function and persistence by providing stimulatory signals for T cells to proliferate [27]. Therefore, we investigated the persistence of DNTs in leukemia-bearing hosts for better resemblance of clinical setting.

**Figure 1:**
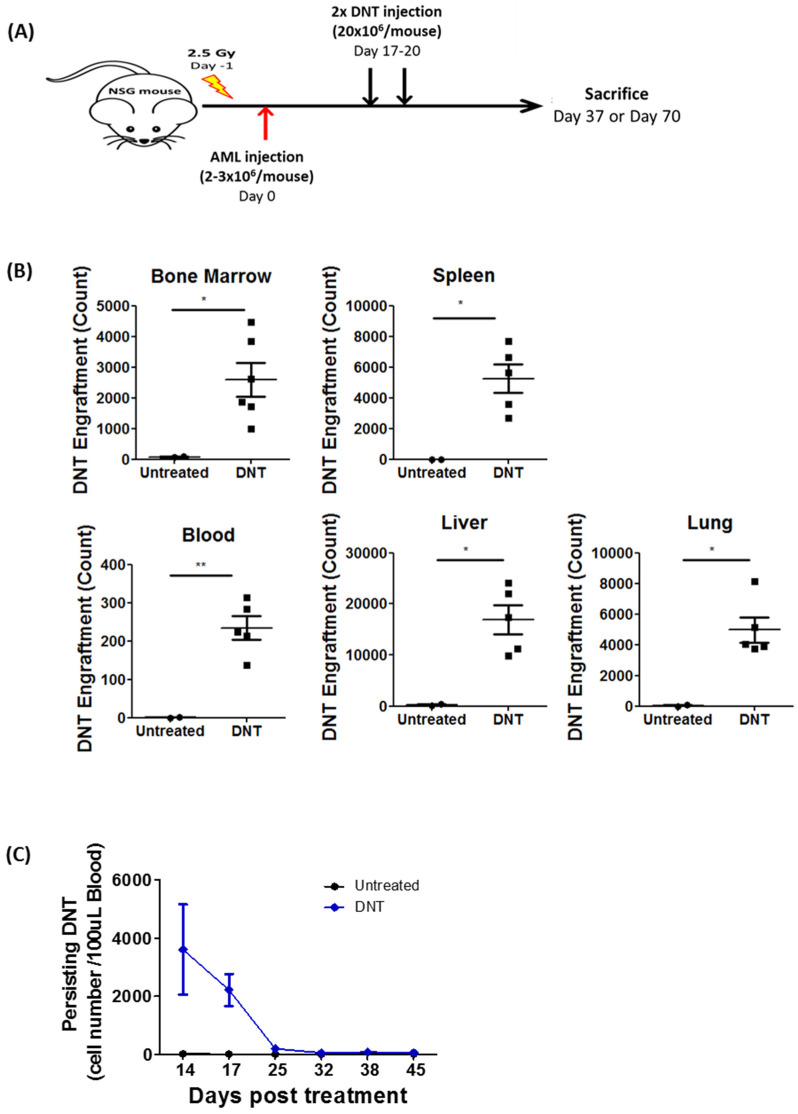

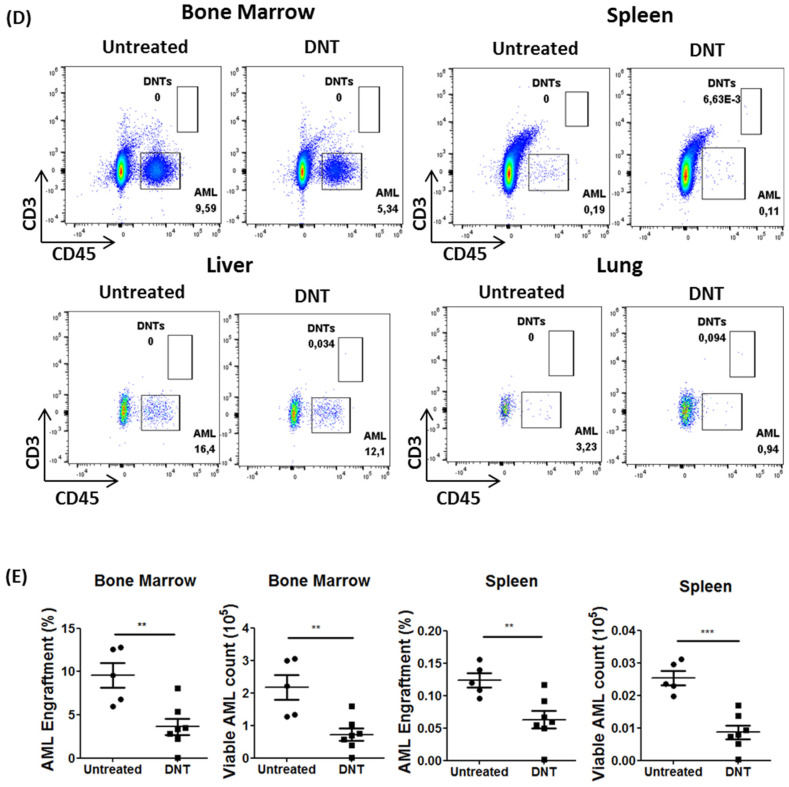
DNT persistence and anti-leukemic activity in leukemia-bearing hosts. (**A**) Schematic diagram of patient-derived xenograft model used. Sublethally irradiated NSG mice were infused with 2–3 × 10^6^ primary AML patient sample on day 0. On day 17 and 20, mice were infused with 2 × 10^7^ DNTs. Mice were sacrificed, and DNT and AML engraftment were assessed in various tissues on day 37 or day 70. (**B**,**C**) NSG mice engrafted with a primary AML sample (090543) were treated with PBS or DNT as described in (**A**). (**B**) Number of viable DNTs engrafted in the bone marrow, spleen, blood, liver, and lung were assessed on day 37, which was 17 days post DNT infusion. (**C**) Number of DNTs detected in mice peripheral blood from day 14 to 45 days post DNT infusion. Each symbol represents individual mice, and error bars represent SEM. (**D**,**E**) NSG mice engrafted with a primary AML sample (130624) were treated with PBS or DNT as described in above. Mice were sacrificed and DNT and AML engraftment were assessed in various tissues on day 70, which was 50 days post-treatment. (**D**) Representative flow plots showing AML engraftment (CD45^low+^ CD3^−^) and DNT engraftment (CD45^high+^ CD3^+^) from untreated and DNT treated groups. (**E**) Dot plot shows frequency and number of viable AML cells in untreated (*n* = 5) and DNT-treated (*n* = 7) mice bone marrow and spleen. Each symbol represents individual mice, the horizontal bars represent the mean, and the error bars represent SEM. The result is representative of two similar experiments with different AML primary blasts. * *p* < 0.05, ** *p* < 0.01, *** *p* < 0.001.

To test this, sublethally irradiated NSG mice were inoculated with primary AML patient blasts followed by infusion of two doses of ex vivo expanded DNTs or PBS. Subsequently, DNT levels were monitored in various tissues (Figure 1A). DNTs were detectable in the BM, spleen, blood, liver, and lung on day 37, which was 17 days post the second DNT treatment (Figure 1B and Figure S1). Further, DNTs were detectable in peripheral blood for up to 25 days post DNT treatment but were no longer detectable after 32 days post DNT treatment, which were day 45 and day 52, respectively (Figure 1C). By day 70, 50 days post-treatment, no DNTs were detectable in the BM with negligible levels of DNTs found in the spleen (0.016 ± 0.004%), liver (0.122 ± 0.054%), and lung (0.23 ± 0.0522%) (Figure 1D). Consistent with our previous report, DNTs significantly reduced the frequency and total number of AML cells in the BM and spleen compared to the PBS-treated group (Figure 1D,E). However, significant levels of residual AML cells were detected with very little to no DNTs persisting (Figure 1D,E). Given that residual AML cells detected after the clearance of DNTs can significantly lessen the significance of DNT-mediated anti-leukemic activity, we next investigated ways to improve DNT persistence in vivo.

### 3.2. Ide Significantly Enhances Anti-Leukemic Function of DNTs in Patient-Derived Xenograft Model

When T cells are activated through T cell receptor (TCR), co-stimulatory molecules, and cytokine receptor engagements, the PI3K pathway is activated predominantly through PI3Kδ. The active PI3K pathway then initiates T cell activation and differentiation that generate effector T cells with robust effector function but with low persistence [28,29]. To improve DNT persistence and its long-term anti-leukemic activity in vivo, we investigated the impact of treating DNTs with an FDA-approved PI3Kδ inhibitor, Ide.

First, DNTs were isolated and expanded as previously described [17] in the presence of increasing concentrations of Ide. Compared to the vehicle control, improved cell yield was observed at the end of expansion in the presence of 0.1–1.0 µM Ide, while DNT expansion was compromised from 3.0–10.0 µM of Ide (Figure S2A). In addition, 1 µM Ide resulted in the highest DNT viability at the end of cell expansion cultures (Figure S2B). Based on these results, 1 µM Ide was used to treat DNTs for the rest of the study.

**Figure 2:**
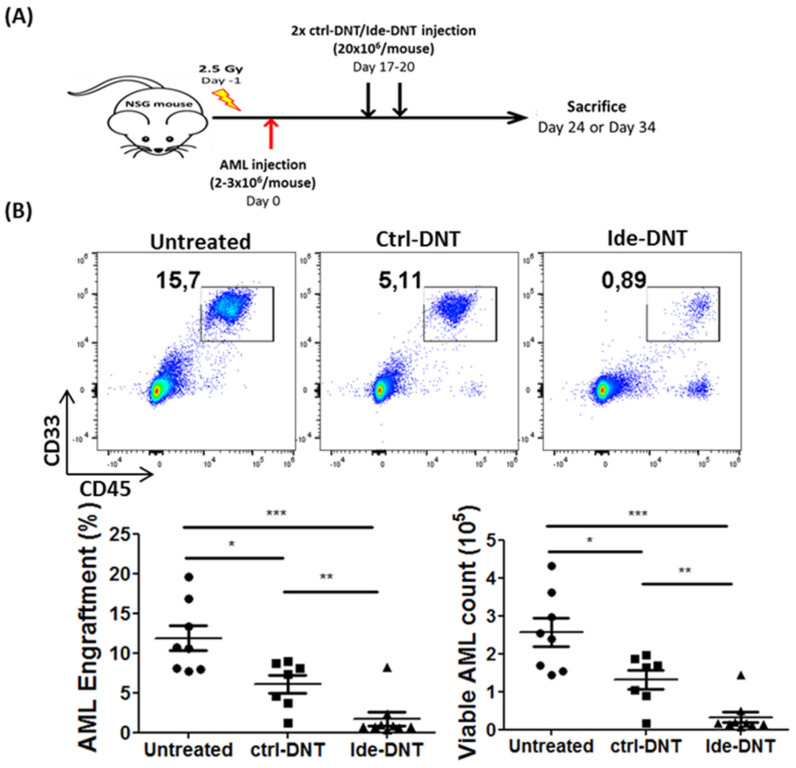

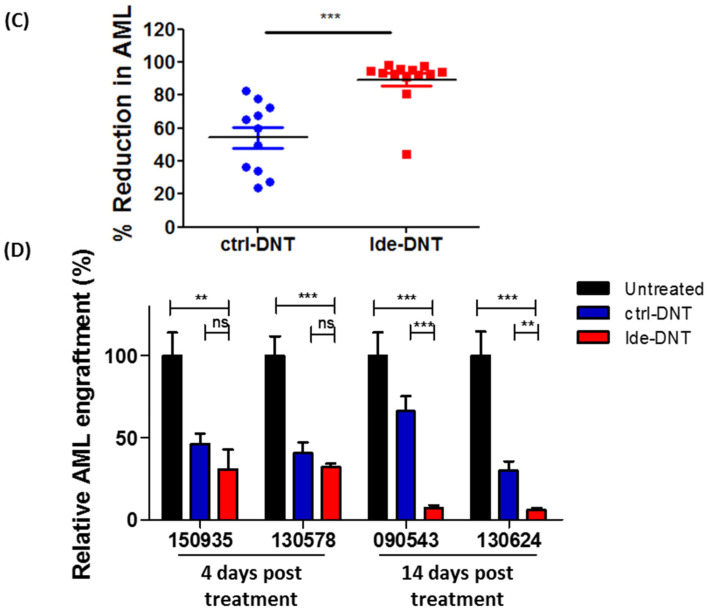
Ide significantly enhances the anti-leukemic function of DNTs in PDX model. (**A**) Schematic diagram of patient-derived xenograft (PDX) model used. Sublethally irradiated NSG mice were infused with 2–3 × 10^6^ primary AML patient sample on day 0. On day 17 and 20, mice were infused with 2 × 10^7^ ctrl-DNTs or Ide-DNTs. Mice were sacrificed and AML engraftment were assessed in various tissues on day 24 or day 34. (**B**) NSG mice engrafted with a primary AML sample (090543) were treated with PBS, ctrl-DNT, or Ide-DNT as described in Figure 2A. AML engraftment and counts in bone marrow were assessed on day 34. Representative flow plots show AML engraftment (CD45^+^ CD33^+^) in the BM. Dot plot shows the frequency and number of AML cells engrafted in the BM. Horizontal line represents the mean of BM AML engraftment level, each symbol represents individual mice, and error bars represent SEM. The results shown are representative of two independent experiments. (**C**) Percent reduction in AML engraftment from two PDX experiments performed using different primary AML patient samples (090543 and 130624). Horizontal bar represents the mean of reduction in AML engraftment level in BM on day 34 relative to the untreated group in ctrl-DNT (blue)- and Ide-DNT (red)-treated groups. Each symbol represents individual mice and error bars represent SEM. (**D**) Sublethally irradiated (250 cGy) NSG mice were engrafted with one of four primary AML samples (sample ID: 150935, 130578, 090543, and 130624; 2–3 × 10^6^ cells/mouse). Subsequently, mice were treated with PBS (black) or 1.5–2 × 10^7^ cells per infusion of ctrl-DNTs (blue) or Ide-DNTs (red) on day 17 and 20. For the PDX model using the sample IDs 150935 and 130578, BM AML engraftment was assessed on day 24, which was 4 days post-treatment. For the sample IDs 090543 and 130624, BM AML engraftment was assessed on day 34, which was 14 days post-treatment. Each group had 4–8 mice. Horizontal bar represents the mean of BM AML engraftment level normalized to the untreated group, and error bars represent SEM. * *p* < 0.05, ** *p* < 0.01, *** *p* < 0.001.

Next, the anti-leukemic activity of Ide-DNTs was investigated using previously established AML patient-derived xenograft (PDX) models [13]. Briefly, NSG mice engrafted with primary AML samples were treated on day 17 and day 20 with PBS, and DNTs expanded in the presence of Ide or in the presence of vehicle control. Subsequently, AML engraftment was assessed on day 24 or day 34, 4 days or 14 days post-treatment, respectively (Figure 2A). Notably, significantly lower AML levels were observed in Ide-DNT-treated mice compared to untreated and ctrl-DNT-treated groups on day 34 (Figure 2B and Figure S3), where AML counts in BM were 16.38 ± 3.53- and 9.6 ± 2.07-fold lower in the Ide-DNT group relative to the untreated and ctrl-DNT-treated groups, respectively. Summarized results from PDX experiments done using primary AML samples from two different patients show 89.44 ± 3.95% reduction in AML engraftment with Ide-DNT treatment versus 54.27 ± 6.35% reduction with ctrl-DNTs (Figure 2C). Interestingly, when AML engraftment level was assessed 4 days post DNT infusion, the Ide-DNT group showed comparable AML engraftment level as the ctrl-DNT group (Figure 2D). In contrast, superior anti-leukemic activity of Ide-DNT relative to ctrl-DNT was detected 14 days post DNT infusion (Figure 2D). This suggests that the superior anti-leukemic activity of Ide-DNT may be attributable to prolongation of its activity instead of improved potency. Importantly, despite the improved cytotoxicity towards leukemic blasts, off-tumor toxicity of DNTs remained undetectable with or without Ide treatment (Figure S4).

### 3.3. Ide Promotes an Early Memory Subset of DNTs That Have Better Proliferative Capacity

To determine the mechanisms by which Ide enhances DNT function, we first assessed the effect of Ide on the cytotoxic activity of DNTs in vitro. Ide-DNT mediated slightly but statistically significantly reduced cytotoxicity against AML cell lines, including OCI-AML3 and MV4–11, and primary AML blasts more than ctrl-DNTs in vitro (Figure S5). The reduced 2 h cytotoxicity (Figure S5), along with the comparable leukemia burden seen in Ide-DNT and ctrl-DNT groups 4 days post DNT infusion (Figure 2D), supported the idea that the superior in vivo activity of Ide-DNTs is not due to elevated robustness of DNT-mediated cytotoxicity against AML.

**Figure 3:**
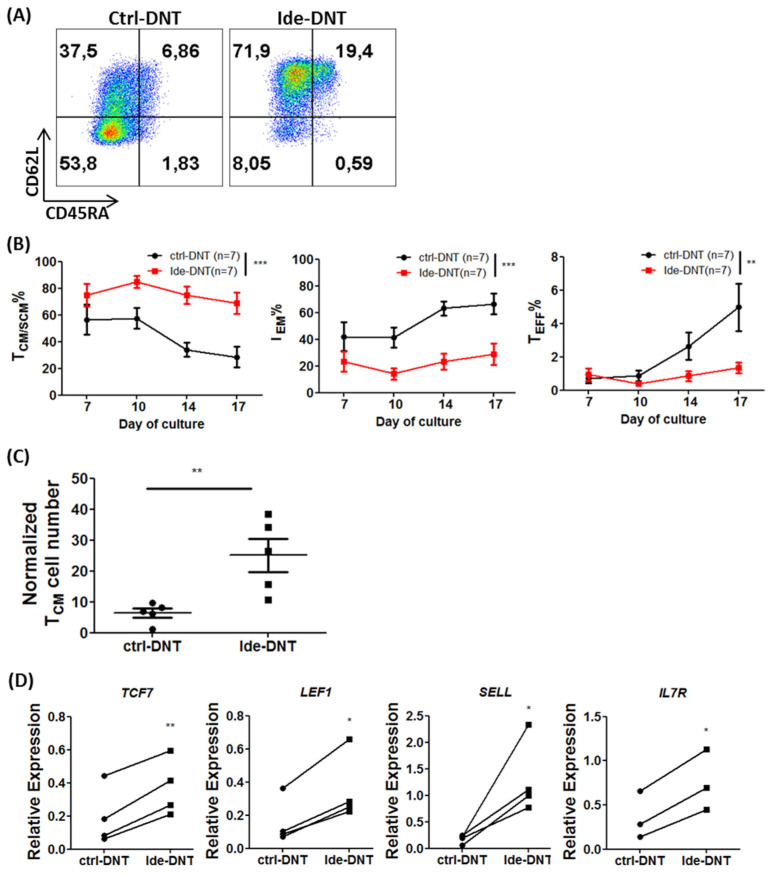

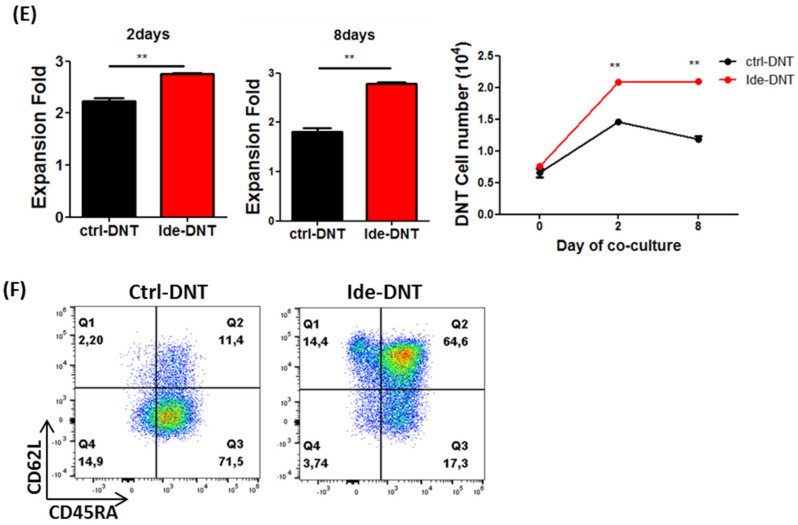
Ide promotes an early memory subset of DNTs that have better proliferative capacity. (**A**) Representative flow plots showing memory status. Memory status of ctrl-DNT and Ide-DNT on day 17 of expansion determined by CD45RA and CD62L expression. T_naive/SCM_ = CD45RA^+^ CD62L^+^; T_CM_ = CD45RA^−^ CD62L^+^; T_EM_ = CD45RA^−^ CD62L^−^; T_effector_ = CD45RA^+^ CD62L^−^. (**B**) Frequency of DNTs in early memory subset T_CM/SCM_ (CD45RA^+^/CD62L^+^ and CD45RA^−^ CD62L^+^; left), T_EM_ (CD45RA^−^ CD62L^−^; middle), and T_EFF_ (CD45RA^+^ CD62L^−^; right) from day 7 to day 17 of ex vivo DNT expansion culture. (**C**) T_CM/SCM_ cell number of ctrl-DNT and Ide-DNT on day 17 of ex vivo DNT expansion culture. Each dot represents DNTs from one donor. The cell number is normalized to day 0 DNT number. (**D**) Relative expression of memory associated genes, *TCF7*, *LEF1*, *SELL*, and *IL7R*, in ctrl-DNT and Ide-DNT determined by qPCR. The expression shown is relative to the expression of housekeeping gene, *HPRT*. Each of the paired symbols represent DNTs from one donor. (**E**) Expansion of ctrl-DNT (black) and Ide-DNTs (red) in the presence of DNT-susceptible AML cell line, OCI-AML3. Expansion fold relative to the number of DNTs at the start of co-culture was determined after 2 days and 8 days (Left). Absolute number of viable DNTs from each treatment group after co-culture with OCI-AML3 (Right). The results shown are representative of three independent experiments. (**F**) Memory status of DNTs 8 days post co-culture with OCI-AML3 determined by flow cytometry. The result shown is representative of three independent experiments. * *p* < 0.05, ** *p* < 0.01, *** *p* < 0.001.

Given that the significant enhancement of anti-leukemic activity of Ide-DNT was observed 14 days, but not 4 days after treatment, we investigated the impact of Ide on DNT memory status. Early memory phenotype of infused cells is an important dictator for immune cell persistence that is linked to a better clinical outcome [30,31]. Notably, the majority of expanded Ide-DNTs exhibited central memory (T_CM_; CD45RA^−^ CD62L^+^) and stem cell memory (T_SCM_; CD45RA^+^ CD62L^+^ CD95^+^) phenotypes, while effector memory (T_EM_; CD45RA^−^ CD62L^−^) DNTs were the dominant subset in ctrl-DNTs (Figure 3A and Figure S6A). A significantly higher proportion of early memory subsets, T_CM_/T_SCM_, and lower frequency of T_EM_ and T_EFF_ populations were observed in Ide-DNTs relative to ctrl-DNTs throughout DNT expansion with more notable differences at a later stage of expansion between day 14 and day 17 (Figure 3B). As a result, there was a 3.87 ± 0.82-fold higher T_CM_/T_SCM_ cell number obtained in the presence of Ide at the end of expansion compared to ctrl-DNTs (Figure 3C). Further, these changes towards the early memory phenotype were observed from Ide-DNTs in a dose-dependent manner (Figure S6B). Consistent with this, stem cell memory-associated transcription factor genes, *TCF7* and *LEF1,* were expressed at a higher level by Ide-DNTs than ctrl-DNTs between day 14 and day 17 of the culture, as well as memory-associated genes, *SELL* and *IL7R* (Figure 3D). However, an insignificant change was observed in *BATF* expression, which encodes a transcription factor important for effector T cell differentiation [32] (Figure S6C).

In addition to genetic and molecular markers, a key biological feature of memory T cells is their ability to persist and proliferate after encountering their targets [33]. Hence, we monitored the proliferative capacity and viable cell counts for Ide-DNTs and ctrl-DNTs during co-culture with an AML cell line, OCI-AML3, for 8 days. We observed a significantly higher degree of proliferation (Figure S7) and number (Figure 3E) of viable DNTs when Ide-DNTs were co-cultured with AML cells than the ctrl-DNT. Importantly, their early memory phenotype was maintained for at least 8 days post co-culture with AML targets, while control DNTs presented a terminally differentiated effector phenotype (Figure 3F). Collectively, these observations demonstrate that Ide promotes an early memory status of DNTs that may contribute to the superior in vivo leukemia control of Ide-DNT.

### 3.4. Ide Improves Fitness of DNTs by Inhibiting Exhaustion and Enhancing Viability

**Figure 4:**
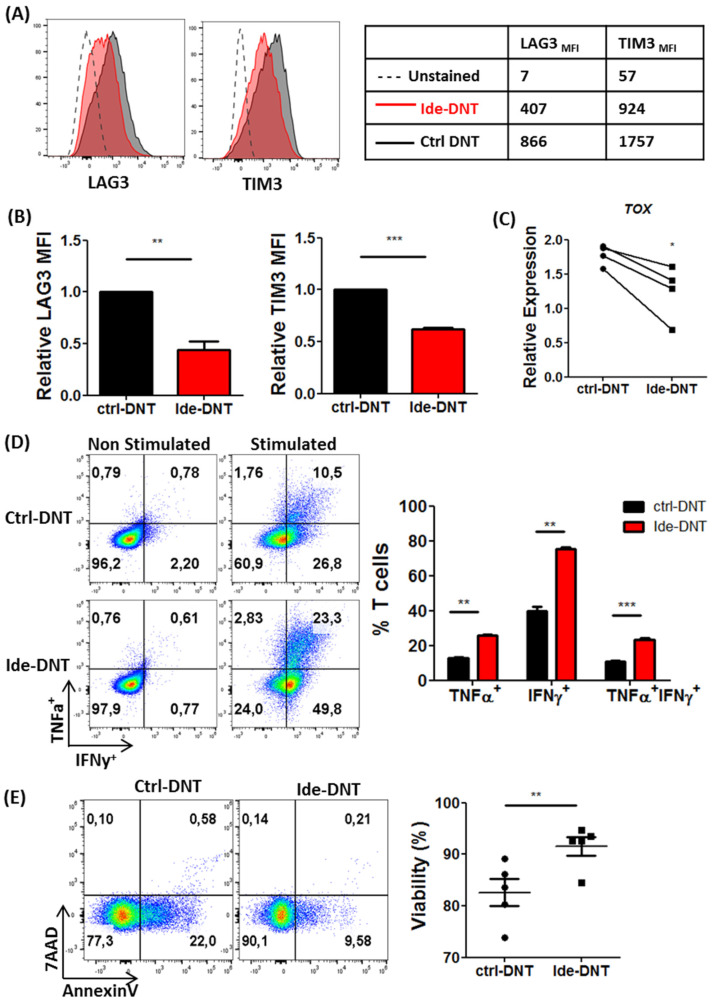

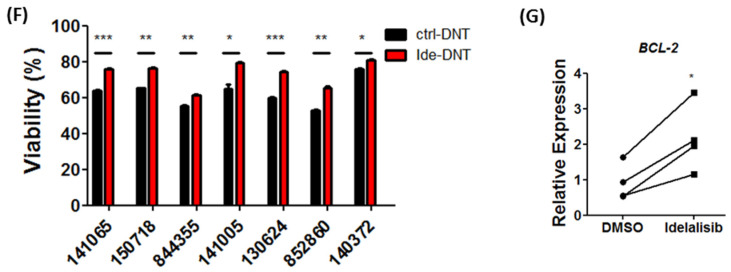
Ide improves fitness of DNTs by inhibiting exhaustion and enhancing viability. (**A**) Expression of exhaustion markers, LAG3 and TIM3, on unstained (dotted line), ctrl-DNT (Red), and Ide-DNT (Black). Representative flow plot is shown (Left). The numbers represent the MFI value (Right). (**B**) The summary of results comparing the relative MFI of LAG3 and TIM3 on the ctrl-DNT and Ide-DNT from five donors. Horizontal bars represent the mean relative MFI and error bars show SEM. (**C**) Expression of the exhaustion-associated transcription factor gene, *TOX*, relative to the expression of the housekeeping gene, *HPRT*, determined by qPCR. Each paired symbol represents DNTs from one individual. (**D**) Production of TNFα and IFNγ by the ctrl-DNT and Ide-DNT with or without ex vivo stimulation. The ctrl-DNT and Ide-DNT were treated with a protein transport inhibitor, followed by stimulation using anti-CD3/CD28 activation beads for 4 h. Representative flow plot is shown (Left). The numbers represent the frequency in each gate. Bar graph shows the percentage of ctrl-DNT (black) and Ide-DNT (red) expressing TNFα, IFNγ, and TNFα and IFNγ (Right). The result shown is a summary of three experiments. (**E**) Frequency of viable ctrl-DNTs and Ide-DNTs on day 17 of expansion. The cell viability was determined with Annexin V and 7-AAD staining using flow cytometry. Flow plot shows a representative image of cell viability (Left). Dot plot shows summary of viability results using DNTs from five different donors (Right). Horizontal bars represent the mean, and the error bar shows SEM. (**F**) Viability of ctrl-DNTs and Ide-DNTs after co-culture with primary AML cells from seven different patients. Ctrl-DNTs and Ide-DNs were co-incubated with primary AML cells at 4:1 effector-to-target ratio for 2 h. Subsequently, the viability of DNTs (gated on CD45^high^ CD3^+^ CD33/CD34^−^) were determined using flow cytometry. (**G**) Expression of the anti-apoptotic gene, *BCL2*, on ctrl-DNT and Ide-DNTs relative to the expression of the housekeeping gene, *HPRT*, was determined by qPCR. Each paired symbol represents DNTs from one donor. * *p* < 0.05, ** *p* < 0.01, *** *p* < 0.001.

Next, the effect of Ide on DNT exhaustion was studied as T cell exhaustion can significantly impair in vivo anti-tumoral activity [30,34]. Ide treatment reduced the expression of inhibitory receptors, Lag3 and Tim3, on DNTs compared to the ctrl-DNTs (Figure 4A,B). Furthermore, Ide-DNTs exhibited significantly lower expression of *TOX,* a transcription factor gene known as the central regulator of T cell exhaustion (Figure 4C). Functionally, Ide-DNTs mediated superior single- (IFNy^+^ or TNFα^+^) and multi-cytokine (IFNy^+^ and TNFα^+^) production after stimulation, further supporting the less exhausted state than control DNTs (Figure 4D).

Further, we observed that Ide significantly improved the viability of DNTs from five healthy donors with reduced apoptotic DNTs after expansion (Figure 4E). Moreover, Ide-DNTs remained more viable after co-culture with primary AML blasts from seven patients (Figure 4F). In agreement with this, higher expression of the anti-apoptotic gene, *BCL-2* was detected from Ide-DNTs compared to ctrl-DNTs (Figure 4G). Collectively, these results demonstrate that Ide prevents exhaustion and improves viability of DNTs, resulting in DNTs with a better fitness at the end of expansion.

### 3.5. Ide Prolongs Persistence of DNTs and Promotes a Durable Anti-Leukemic Effect

**Figure 5:**
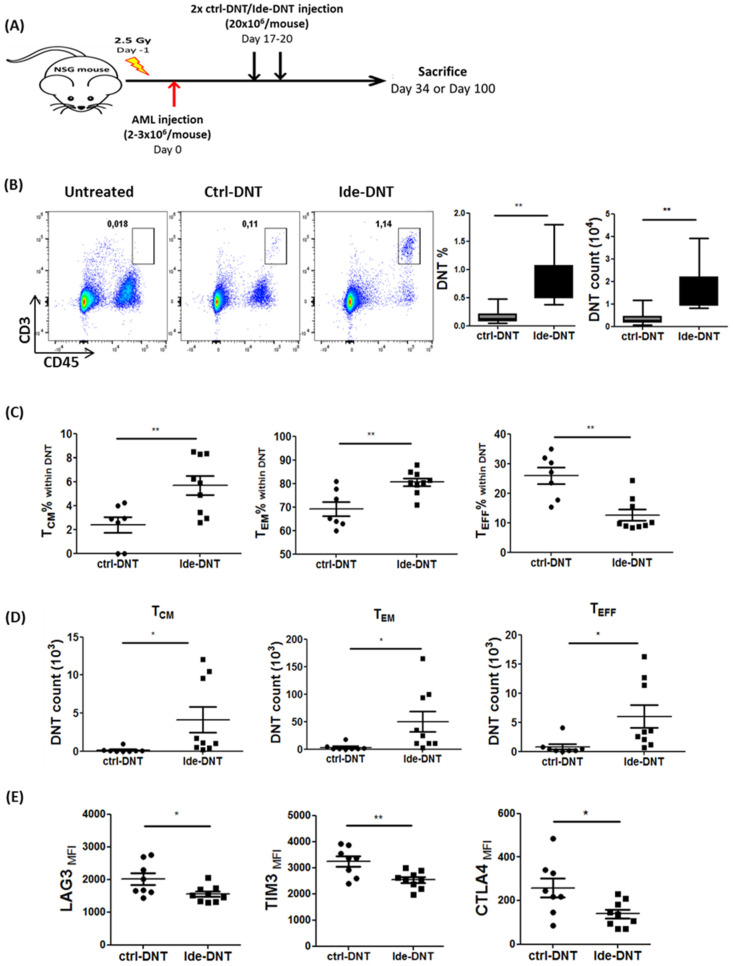

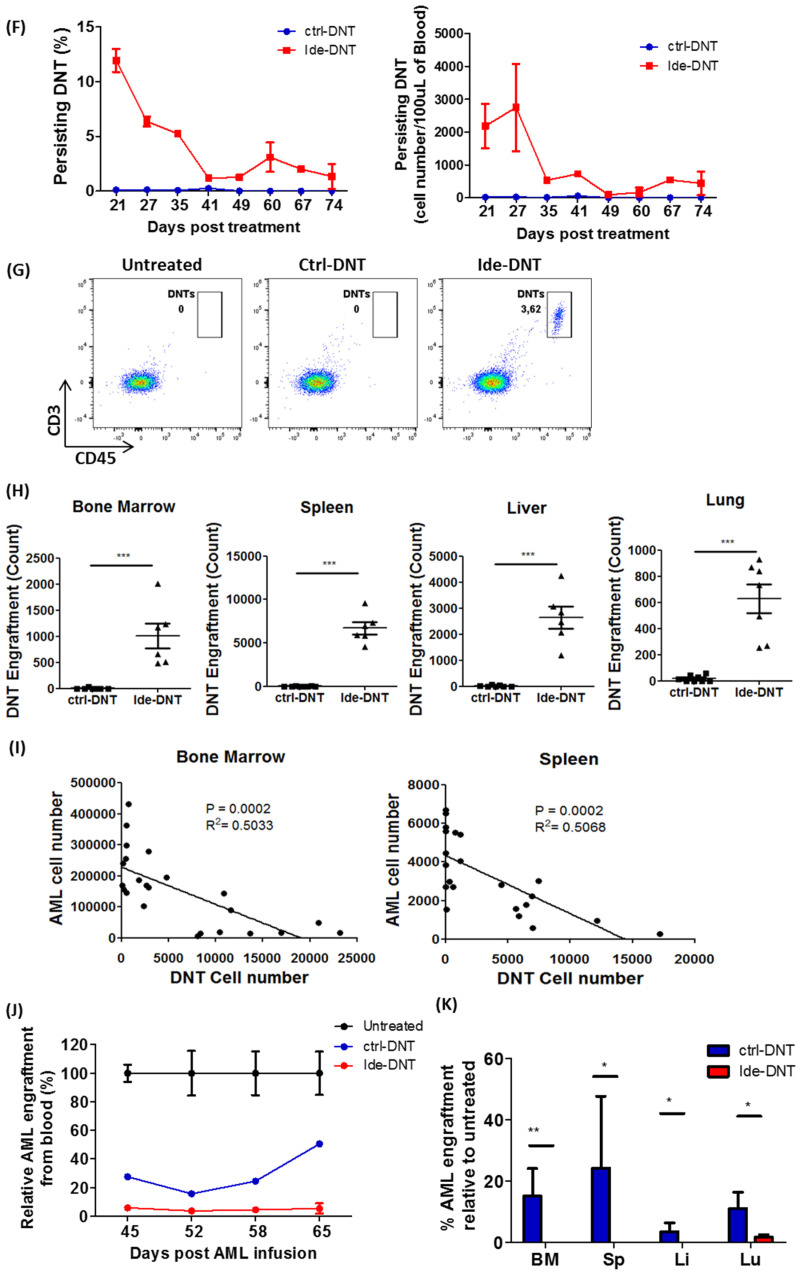

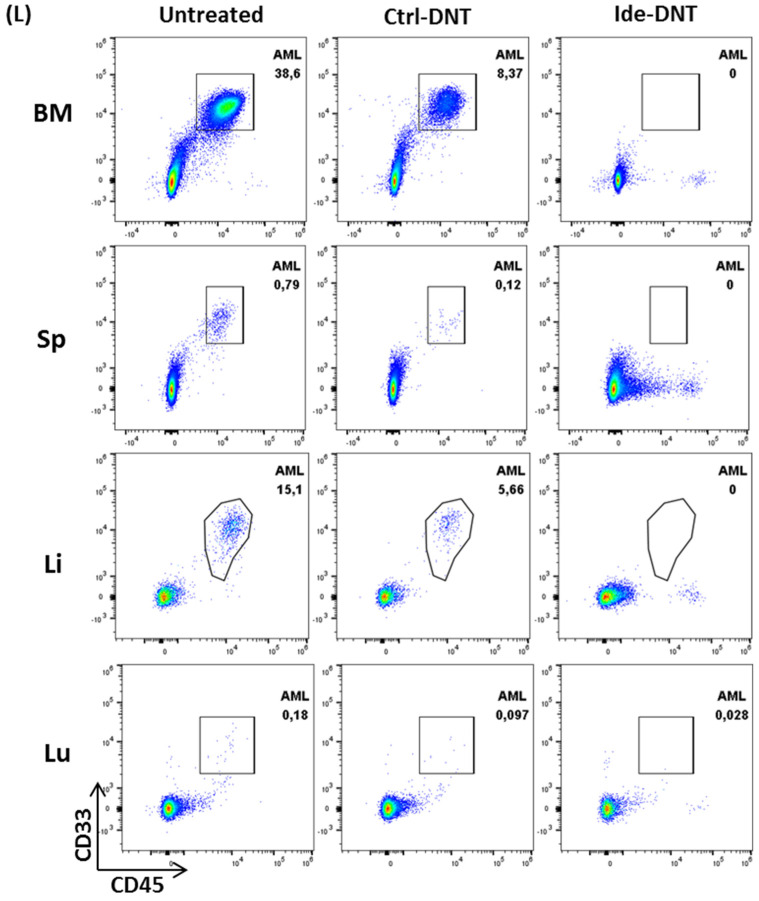
Ide prolongs persistence of DNT and promotes a durable anti-leukemic effect in vivo. (**A**) Schematic diagram of patient-derived xenograft (PDX) model used. Sublethally irradiated NSG mice were infused with 2–3 × 10^6^ primary AML patient sample on day 0. On day 17 and 20, mice were infused with 2 × 10^7^ ctrl-DNTs or Ide-DNTs. Mice were sacrificed, and DNT persistence and AML engraftment were assessed in various tissues on day 34 or day 100. (**B**–**E**) NSG mice engrafted with the primary AML sample (090543) were treated with PBS (*n* = 8), ctrl-DNT (*n* = 8), or Ide-DNT (*n* = 9), on day 17 and 20 post AML infusion. Subsequently, (**B**) DNT engraftment in BM (CD45^high^ CD3^+^), (**C**) the frequency, (**D**) and the number of T_CM_ (CD45^high^ CD3^+^ CD45RA^−^ CD62L^+^), T_EM_ (CD45^high^ CD3^+^ CD45RA^−^ CD62L^−^), and T_EFF_ (CD45^high^ CD3^+^ CD45RA^+^ CD62L^−^), as well as (**E**) the expression level of the exhaustion markers, LAG3, TIM3, and CTLA4, within the DNT population were determined on day 34, which was 14 days post-treatment using flow cytometry. Each dot represents one mouse. The result is representative of 2 similar experiments with different AML primary blasts. (**F**–**H**) Sublethally irradiated NSG mice were engrafted with primary AML sample (090517), followed by PBS, ctrl-DNT, or Ide-DNT treatment on day 17 and 20. (**F**) Subsequently, the frequency (left) and number (right) of DNTs in peripheral blood were determined between day 21–74 post last DNT infusion in ctrl-DNT and Ide-DNT using flow cytometry. (**G**) Representative flow plots show DNT persistence from peripheral blood on day 94, which was 74 days post-treatment. (**H**) Number of viable DNT engrafted in the bone marrow, spleen, blood, liver, and lung on day 100, which was 80 days post-treatment. Each dot represents one mouse. (**I**) Correlative analysis of DNT and AML cell numbers in the bone marrow (left) and spleen (right) in AML-engrafted mice treated with ex vivo expanded DNTs. The results are representative of three independent xenograft experiments done using AML cell line, MV4-11 (*n* = 1), and two primary AML patient samples. (**J**) NSG mice engrafted with the primary AML sample (090543) were treated with PBS, ctrl-DNT, and Ide-DNT on day 17 and 20. Subsequently, the AML level in mice peripheral blood relative to the untreated group was determined on day 45, 52, 58, and 65. (**K**,**L**) NSG mice engrafted with the primary AML sample (090517) were treated with PBS (*n* = 5), ctrl-DNT (*n* = 7), and Ide-DNT (*n* = 6) on day 17 and 20. (**K**) Subsequently, % AML engraftment relative to untreated were determined on day 100, which was 80 days post-treatment. (**L**) Representative flow plots show AML engraftment (CD45^low+^ CD33^+^) from the BM, spleen, liver, and lung. * *p* < 0.05, ** *p* < 0.01, *** *p* < 0.001.

Given that Ide skews DNTs towards various characteristics associated with improved persistence of immune cells, such as early memory status, improved proliferative capacity, and reduced exhaustion, we monitored the distribution and persistence of Ide-DNTs and ctrl-DNTs using the PDX model described above (Figure 5A). Compared to the ctrl-DNT group, not only did Ide-DNTs show improved persistence in lymphoid organs like the BM (Figure 5B) and spleen (Figure S8A), but we also observed a significantly increased number of DNTs in non-lymphoid organs like the liver (Figure S8B) on day 34, which was 14 days post DNT infusion. Furthermore, Ide-DNTs detected in xenografted mice were significantly less differentiated than ctrl-DNTs with a higher proportion of T_CM_ and T_EM_ subsets (Figure 5C). In contrast, a higher frequency of persisting ctrl-DNTs was observed in the terminally differentiated effector subset (T_EFF_) (Figure 5C). Moreover, a 27.46 ± 11.13- and 7.57 ± 2.45-fold increase in the total number of DNT_CM_ and DNT_EM_, respectively, was observed in the Ide-DNT group relative to the ctrl-DNT group (Figure 5D). Interestingly, while DNT_EFF_ frequency was lower, the total number of DNT_EFF_ was 15.85 ± 5.91-fold higher in the Ide-DNT group compared to ctrl-DNT group (Figure 5C,D). Consistent with the in vitro observation, Ide-DNTs were less exhausted than ctrl-DNTs (Figure 5E).

Next, we studied the long-term persistence of DNTs by monitoring the DNT levels in blood up to 74 days post DNT infusion (Figure 5F). A significantly higher number and percentage of DNTs were detected in blood from day 21 to day 74 post DNT infusion in Ide-DNT-treated mice compared to ctrl-DNT-treated ones (Figure 5F,G). In addition, DNTs were detectable in the Ide-DNT group in various organs including the BM, spleen, liver, and lung at least up to 80 days post-treatment, which was on day 100, but not in the ctrl-DNT group (Figure 5H and Figure S9). These results collectively support the significantly improved engraftment and persistence of DNTs when expanded in the presence of Ide.

Lastly, we studied whether the prolonged persistence of Ide-DNTs led to not only superior, but also more durable anti-leukemic activity compared to ctrl-DNTs. Notably, the significant inverse correlations were observed between the number of AML cells and DNTs in BM and spleen on day 34, where higher DNT counts correlated with lower AML engraftment, demonstrating the importance of DNT persistence in leukemic clearance (Figure 5I). Similarly, AML cells were undetectable in the peripheral blood of Ide-DNT-treated mice from day 45 while detectable levels of peripheral AML blasts of ctrl-DNT-treated mice were found on day 45, albeit at a lower level than the untreated group (Figure 5J). Further, while the peripheral AML counts of Ide-DNT-treated mice remained undetectable up to day 65, the peripheral AML counts of ctrl-DNT-treated mice increased from day 52 to day 65 (Figure 5J). In another PDX experiment conducted with patient AML blasts that migrate systemically, Ide-DNTs eradicated AML cells from both lymphoid (BM and spleen) and non-lymphoid (liver and lung) organs and kept mice in a leukemia-free state for at least 100 days (Figure 5K,L). In contrast, AML cells were detected from all organs of ctrl-DNT-treated mice, although at lower levels than the untreated group (Figure 5K,L). Collectively, these results support the notion that Ide enhances the potency and durability of DNT-mediated leukemia clearance by enhancing the overall fitness and persistence of expanded DNTs.

## 4. Discussion

Previously, we demonstrated the therapeutic potential of DNTs as an off-the-shelf therapy for AML patients [12,13,17]. A recently completed phase I/IIa clinical trial assessed the activity of allogeneic DNTs in AML patients with relapsing disease after allo-HSCT and supported the feasibility, safety, and potential efficacy of allogeneic DNTs for this patient population associated with very poor prognosis [18]. While the above study monitored the total DNT levels in the treated patients, the level and persistence of donor-derived DNTs were not examined. Recent studies reported that ex vivo expanded human DNTs can persist for around 14–21 days in naïve mice [17,26]. However, DNT persistence in leukemia-bearing hosts, which provides more clinically relevant settings, was never studied. In this study, we investigated the persistence of ex vivo expanded DNTs in leukemia-bearing hosts and demonstrated that expanding DNTs in the presence of Ide can significantly improve their persistence, therapeutic efficacy, and durability of response against AML in PDX models.

Consistent with the previous report, ctrl-DNTs mediated a significant anti-leukemic effect in PDX models. The ctrl-DNTs were detectable in blood up to 25–32 days post DNT infusion in leukemia-bearing hosts and in the spleen, liver, and lung up to 50 days post DNT infusion, although the detectable level was very low. The prolonged persistence in leukemia-bearing hosts relative to naïve hosts suggests that the leukemic cells may provide stimulatory signals to DNTs to promote their persistence in vivo. However, the AML level increased starting on day 52, at which time no significant number of DNTs was detected in the peripheral blood of ctrl-DNT-treated mice, suggesting the lack of persistence of ctrl-DNTs may be a limiting factor for a durable anti-leukemic response.

PI3Kδ plays a central role in initiating clonal expansion and differentiation of activated T cells to develop effective anti-tumour activity [29,35,36]. However, the fine balance of activation signal is important to minimize terminal differentiation and exhaustion of anti-leukemic T cells [37,38,39]. Given the strong correlation between the activation and exhaustion status of T cells and their in vivo persistence, we used a PI3Kδ inhibitor, Ide, to overcome the hurdle of limited in vivo persistence and durability of response of DNTs. Notably, Ide slightly reduced potency of DNTs against AML blasts in vitro on a per cell basis, and anti-leukemic activity in ctrl-DNT and Ide-DNT groups were comparable when assessed 4 days post-treatment. However, a significant improvement of anti-leukemic activity was observed in Ide-treated DNTs compared to ctrl-DNTs when assessed at least 14 days post-treatment. Collectively, these results suggest that the superior anti-leukemic responses seen with Ide-DNT are more likely due to improved persistence of DNTs rather than enhancing their cytotoxicity.

Mechanistically, Ide skewed DNTs towards T_CM_ and T_SCM_ subsets with increased expression of stem cell memory-associated genes in Ide-DNTs versus ctrl-DNTs. Blocking of PI3Kδ with Ide significantly dampened DNT activation signal as shown from reduced levels of CD69 expression in a dose-dependent manner (Figure S10). However, lowering the amount of anti-CD3 antibody given during expansion did not recapitulate the memory and exhaustion status seen with Ide (Figure S11A,B), although it significantly reduced the cytotoxicity function against AML (Figure S11C). This finding suggests that Ide-mediated enhancement of DNT persistence is not simply due to the dampening of TCR signalling. In addition, it should be noted that Ide concentrations higher than 1uM significantly compromised the expansion rate of DNTs (Figure S2A). This suggests rather than disposing PI3Kδ signalling, a fine balance of the PI3K activation pathway is required to warrant optimal DNT expansion.

T cell exhaustion affects the proliferative capacity as well as cytotoxicity activity of T cells against tumours, resulting in poor persistence and overall outcomes [30,34]. PI3Kδ blockade reduced the exhaustion level of DNTs, leading to superior proliferative capacity and ability to produce effector cytokines (IFNγ and TNFα) upon stimulation. Moreover, a lower expression of transcription factor TOX was detected. However, further investigation is needed to determine if the lower TOX expression seen in Ide-DNT is responsible for the less exhausted state of DNTs. A recent study reported Regnase-1, a ribonuclease that regulates immune cell activation, as a crucial negative regulator for programming high *Lef1* and *Tcf7* but low *Tox*-expressing CD8^+^ T cells by suppressing BATF [40,41]. Although lower expression of *TOX* and a high expression of *LEF1* and *TCF7* was observed in Ide-DNTs, comparable levels of *REGNASE-1* and *BATF* were observed between Ide-DNTs and ctrl-DNTs, suggesting that the Ide-mediated effect is Reganse-1 independent (Figure S6C and Figure S12).

Ide is an FDA-approved drug for treatment of lymphoma including chronic lymphocytic leukemia, follicular B-cell non-Hodgkin lymphoma, and small lymphocytic leukemia [42,43]. However, severe adverse events associated with Ide treatment have been reported including autoimmune toxicities, infection, diarrhea, and skin problems [43,44,45] as PI3Kδ blockade interrupts T cells homeostasis and suppresses Treg proliferation and function [46,47,48]. Given the reported side effects of Ide, DNTs were pre-treated with Ide ex vivo during expansion and washed prior to in vivo infusion. Similar to our observation, other groups have shown that expansion of CAR-transduced CD4^+^ and CD8^+^ T cells with Ide can improve their in vivo persistence and anti-tumoral activity by promoting memory phenotype and reducing T cell exhaustion [49,50,51]. This suggests that, while DNTs is a rare subset of T cell with distinct mechanisms of anti-leukemic activity, it shares similarity in the PI3K-dependent activation signalling with conventional T cells.

Our study collectively highlights that Ide treatment improves the overall fitness of DNTs to promote better engraftment and longer persistence in vivo. Further, this study was the first to investigate the persistence of DNTs and the first to show the correlation between DNT persistence and the durability of the treatment in vivo, emphasizing the importance of DNT persistence. We and others have previously reported that DNTs can target an array of tumours including haematological and solid tumours in vivo [13,14,15]. Although this study primarily focused on investigating the effect of Ide on DNTs’ function against AML, our data suggest that Ide may also enhance therapeutic efficacy against other tumours by enhancing the overall fitness of DNTs and improving their in vivo persistence. Further, given that the clinical applications of both Ide and DNTs have been tested, the translation of the findings from this study into the clinic can be accelerated, where DNTs used in clinical trials can be expanded in the presence of Ide for enhanced efficacy and durability of response.

## 5. Conclusions

In this study we investigated the persistence of DNTs in leukemia-bearing mice, providing clinically more relevant settings than in naïve mice. DNTs persistence was observed up to 50 days in various tissues from leukemia bearing hosts, although the detectable level was very low. Further, it was the first to investigate the effect of Ide on DNTs, demonstrating feasible and effective method to improve their persistence and therapeutic efficacy in vivo. Ide treatment of healthy donor-derived DNTs promoted early memory subsets and improved overall fitness by reducing exhaustion while improving viability. These Ide-induced attributes led to prolonged persistence of DNTs leukemia-bearing hosts. Further, Ide-treated DNTs not only improved the anti-leukemic efficacy but also enhanced the durability of the treatment in vivo. Collectively, our study highlights the importance of DNT persistence and Ide mediated improvements in the overall fitness of DNTs to promote better engraftment and longer persistence in vivo. Given that clinical application of both Ide and DNTs have been tested, translation of the findings from this study into clinic can be accelerated, where DNTs used in clinical trials can be expanded in the presence of Ide for enhanced efficacy and durability of response.

## Data Availability

Not applicable.

## Supplementary Materials

The following are available online at https://www.mdpi.com/article/10.3390/cancers13205039/s1, Table S1: Clinical data of primary AML samples. Table S2: List of primer sequences used for Real-Time PCR. Figure S1: DNT persistence in leukemic bearing mice. Figure S2: Expansion of DNTs in the varying concentrating of Ide. Figure S3: Ide treated DNTs significantly reduce AML engraftment level in spleen. Figure S4: ctrl-DNTs and Ide-DNTs do not cause tissue damage. Figure S5: Ide treatment reduce anti-leukemic function of DNTs in vitro. Cytotoxicity, Figure S6: Ide promotes early memory subset of DNTs. Figure S7: Ide enhances proliferative capacity of DNT after encountering AML in vitro. Figure S8: Idelalisib prolongs persistence of DNTs in vivo. Figure S9: Ide prolongs the persistence of DNT. Figure S10: Idelalisib inhibits activation of DNTs.
