# Supplementary material for: Enhancing Therapeutic Efficacy of Double Negative T Cells against Acute Myeloid Leukemia Using Idelalisib
Source: Cancers (Basel). 2021 Oct 9;13(20):5039. doi: 10.3390/cancers13205039 (PMC8533698; doi:10.3390/cancers13205039)
Supplement: Supplementary file 1 [file cancers-13-05039-s001.zip › cancers-1399918-supplementary.pdf]

cancers

MDPI

Supplementary Materials

Enhancing Therapeutic Efficacy of Double Negative T cells against Acute Myeloid Leukemia using Idelalisib

Hyeonjeong Kang, Jong Bok Lee, Ismat Khatri, Yoosu Na, Cheryl D'Souza, Andrea Arruda, Mark D. Minden and Li Zhang

Table S1. Clinical data of primary AML samples.

Sample I.D.	Age	Sex	Cytogenetics	FAB	MRC
90543	34	M	46,XY,inv(3)(q21q26.2),t(9;22)(q34;q11.2) [9]/46,XY [1]	M2	adverse
150935	21	M	46,XY,inv(16)(p13.1q22)[5]/46,XY [5]	M4Eo	favorable
130578	62	M	46,XY [20]	M4	favorable
130624	79	M	46,XY [20]	n.d.	Intermediate(normal)
90517	62	M	46,XY [20]	M1	Adverse (FLT3-ITD+)
141065	57	M	46,XY [20]	n.d.	Intermediate (normal)
150718	58	M	46,Y,t(X;14)(q26;q22),t(2;3)(p25;q25),t(3;15)(q26; q11.2),del(13)(q12q22),add(17)(p13) [11]	n.d.	Adverse
844355	83	F	46,XX [20]	n.d.	Intermediate (normal)
852860	69	F	43~45,XX,add(3)(p25),-4,-5,i(5)(p10),-7,add(9)(p24),der(16)t(16;17)(p11.2;q11.2),t(16;17)(p11.2;q11.2),-17,+1~3mar[cp9]/46,XX[1]	n.d.	Adverse
140372	73	M	46,XY [20]	M4	Adverse

Table S2. List of primer sequences used for Real-Time PCR.

Gene	Sense	Anti-Sense
LEF-1	AGACAAGCACAAACCTCTCAG	TCATTATGTACCCGGAATAACTCG
IL7R	CGCCAGGAAAAGGATGAAA	ATACATTGCTGCCGGTTGG
SELL	GGAATCTGGTCAAATCCTAGTCC	AGCCAAATGATAAATGCCAACC
BATF	GACAGAGGCAGACACAGAAG	TGCTTGATCTCCTTGCGTAG
REGNASE-1	TTCCTGCGTAAGAAGCCACT	AATCGGCACTTGATCCCATA
BCL-2	GTGGATGACTGAGTACCTGAAC	GCCAGGAGAAATCAAACAGAGG
TCF-7	GTCTACTCCGCCTTCAATCTG	GTGGGCTGTTGAAATGTTCG
TOX	CCACCACTCACCATCTCCAC	TTTTCCCCATATCAGAGGCAG

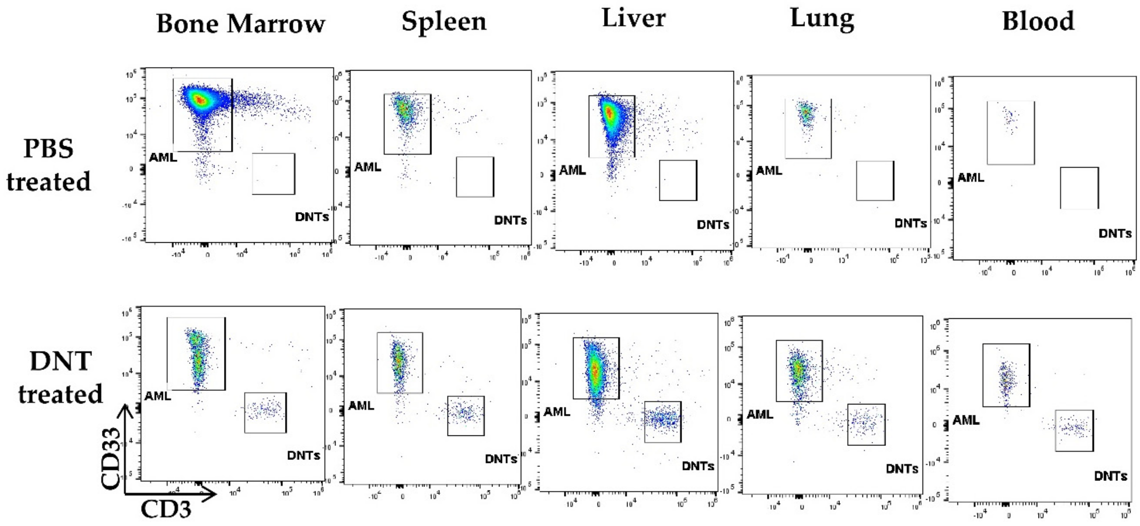

Figure S1. DNT persistence in leukemic bearing mice. NSG mice engrafted with a primary AML sample (090543) were treated with PBS (top panels) or DNT (bottom panels) as described in Figure 1A. Cells are gated within DAPI$^-$ CD45$^+$. Representative flow plots showing AML (DAPI$^-$CD45$^+$CD3$^-$CD33$^+$) and DNT (DAPI$^-$CD45$^+$CD3$^+$CD33$^-$) engraftment from untreated or DNT treated group on day 37, which was 17 days post DNT infusion.

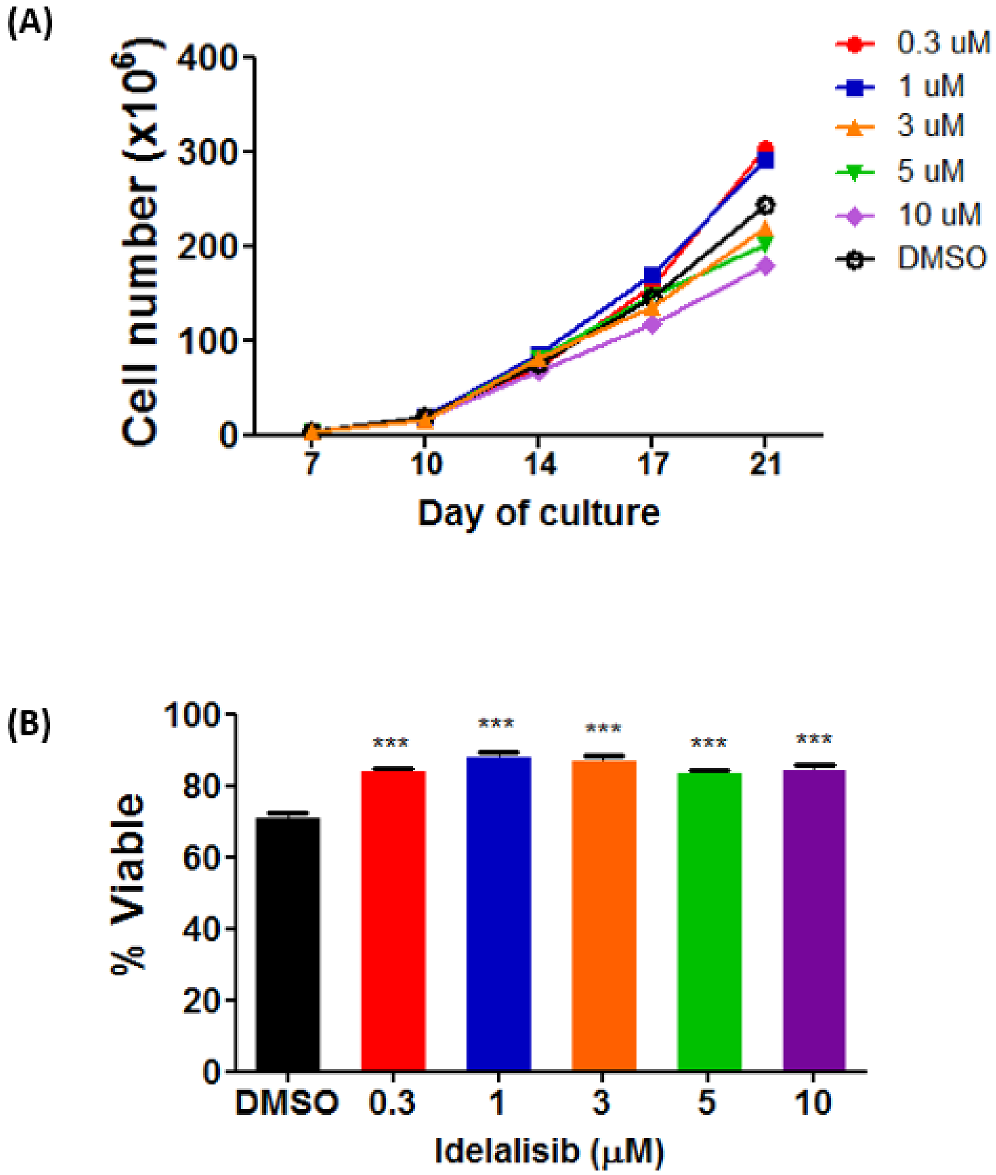

Figure S2. Expansion of DNTs in the varying concentrating of Ide. (**A** and **B**) DNTs expanded in varying concentration of Ide (0.3-10 μM) or vehicle control from day 0 to day 21. Cell number of DNTs from day7 to day 21 of ex vivo DNT expansion culture (**A**). % viable (FSC/SSC$_{\text{live lymphocyte}}$ × AnnexinV^{-}) DNTs on day 21 (**B**).

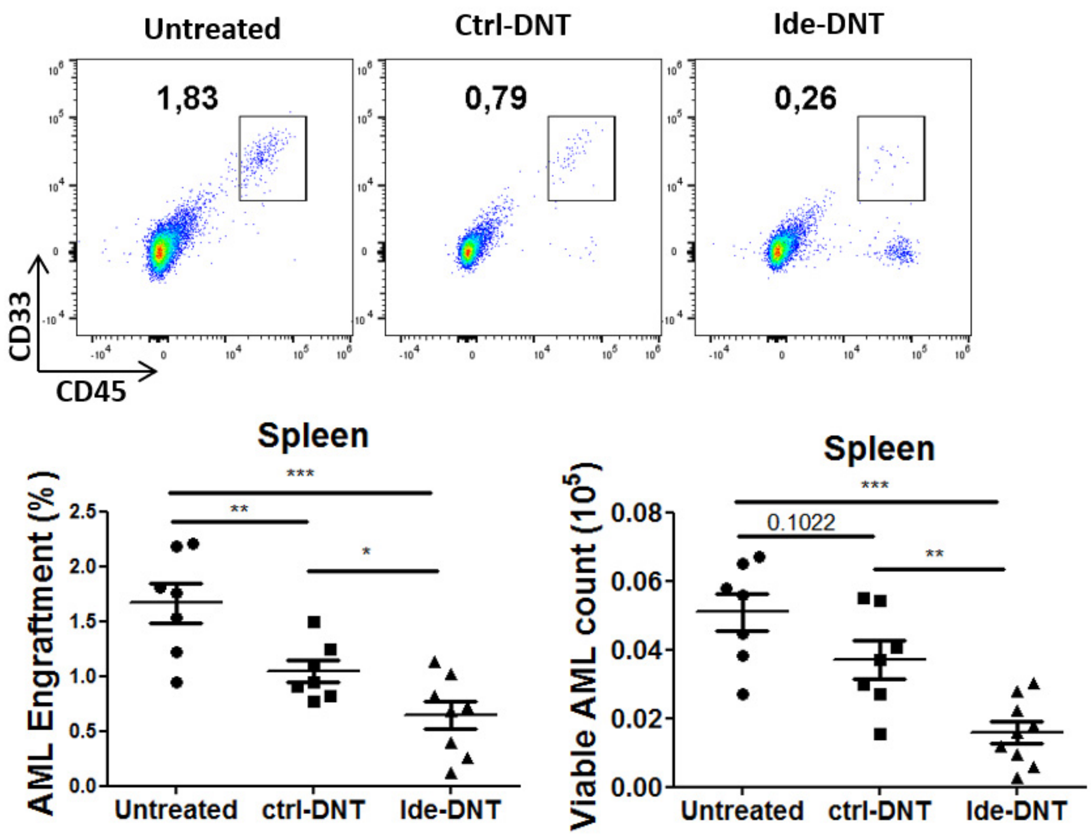

Figure S3. Ide treated DNTs significantly reduce AML engraftment level in spleen. NSG mice engrafted with a primary AML sample (090543) were treated with PBS, ctrl-DNT or Ide-DNT as described in Figure 1A. AML engraftment and counts in spleen were assessed on day 34. Representative flow plots show spleen AML engraftment ($CD45^{+}CD33^{+}$; Top). Dot plot shows the frequency and number of AML cell engrafted in the spleen (Bottom). Horizontal line represents the mean of AML engraftment level and each symbol represents individual mice, and error bars represent SEM. The results shown are representative of two independent experiments.

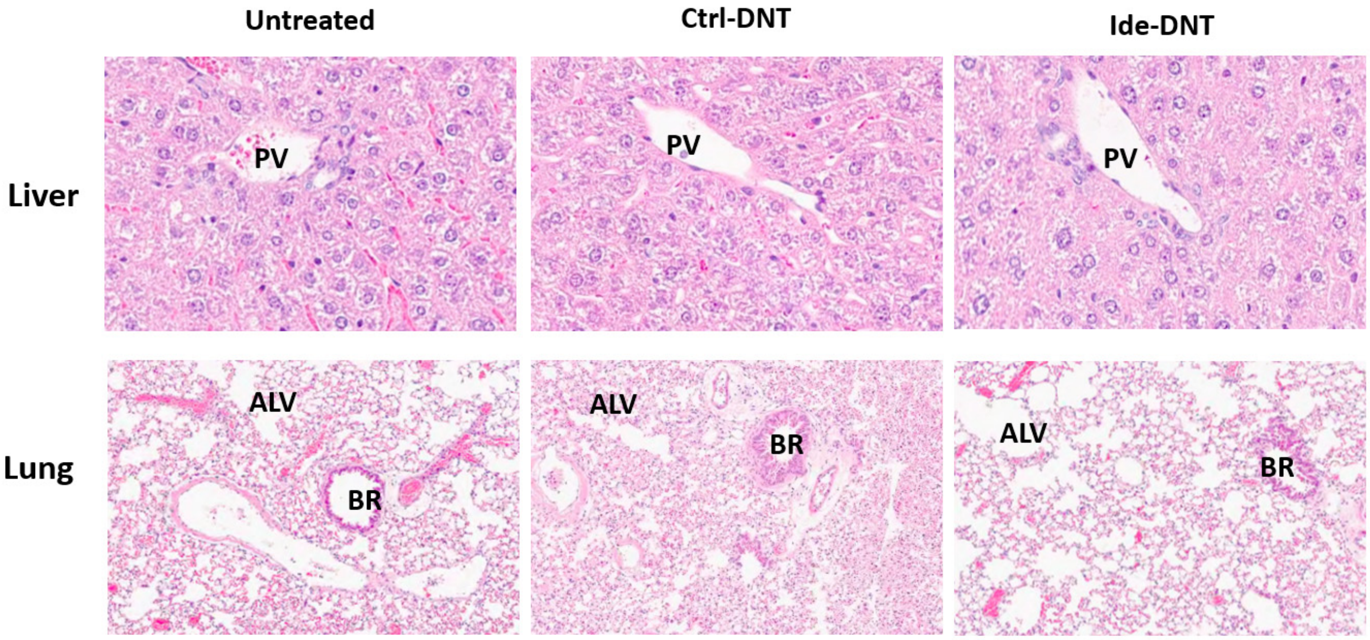

Figure S4. ctrl-DNTs and Ide-DNTs do not cause tissue damage. NSG mice engrafted with primary AML sample (090543) were treated with PBS (n = 8), ctrl-DNT (n = 8), or Ide-DNT (n = 9) on day 17 and 20 post AML infusion. Subsequently, tissue from liver and lung were fixed on day34 for H&E staining. Representative H&E-stained slides of liver (Top) with ×400 magnification and lung (Bottom) with ×100 magnification from untreated (Left), ctrl-DNT(Middle) and Ide-DNT(Right) group. PV-portal vein; ALV-alveoli; BR-bronchioles.

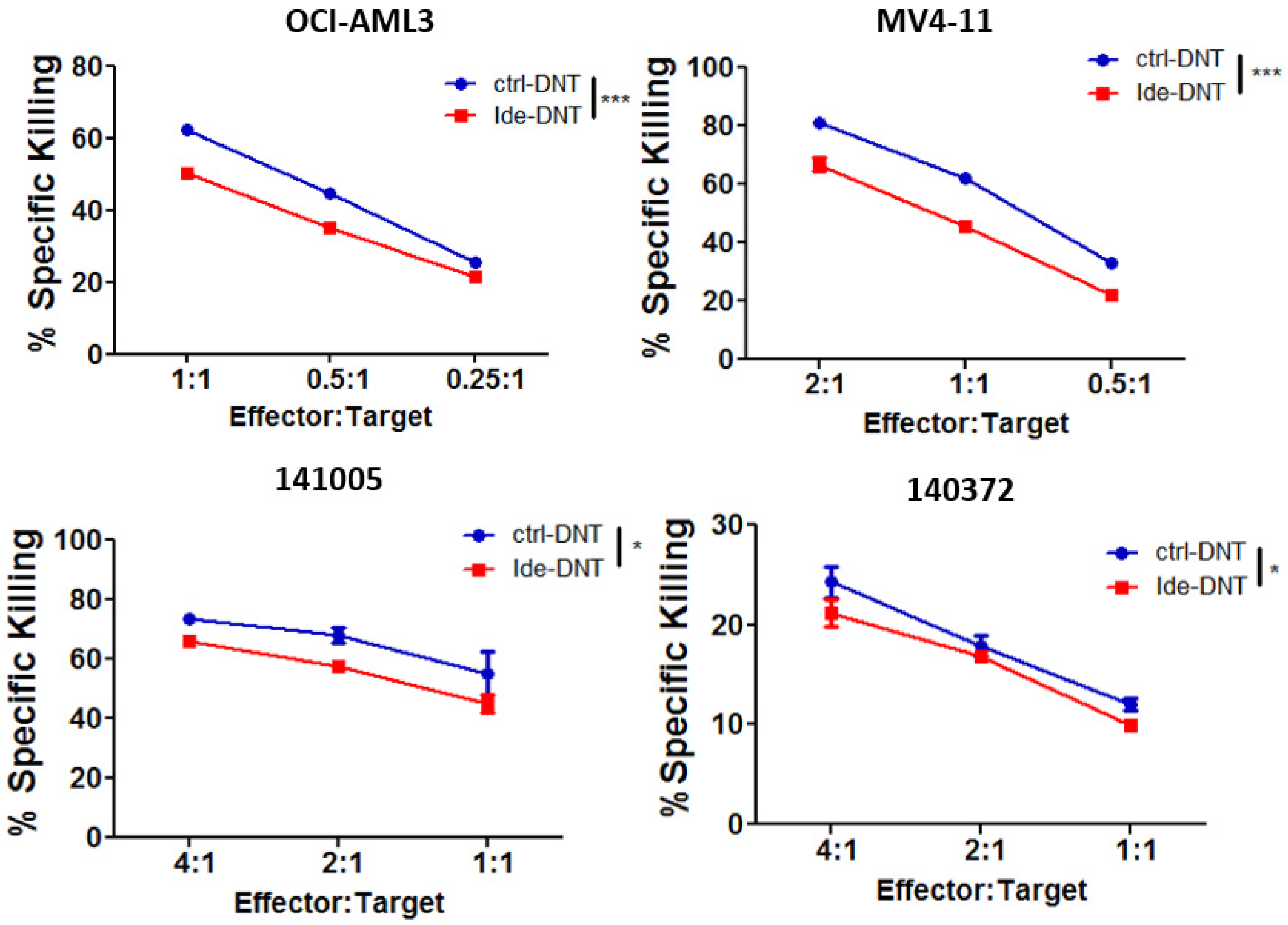

Figure S5. Ide treatment reduce anti-leukemic function of DNTs in vitro. Cytotoxicity Assay with ctrl-DNT or Ide-DNT as effector cells and AML cell lines, OCI-AML3 and MV4-11, or primary blasts, 141005 and 140372, as target cells. 14–17 days ex vivo expanded ctrl-DNT and Ide-DNT were co-cultured with target cells for 2 hours. Subsequently, cells were stained with Annexin V, CD3, CD33/CD34. Viability of targets (gated on $CD3^-$ $CD33/CD34^+$) was determined using flow cytometry to calculate % specific killing by DNTs.

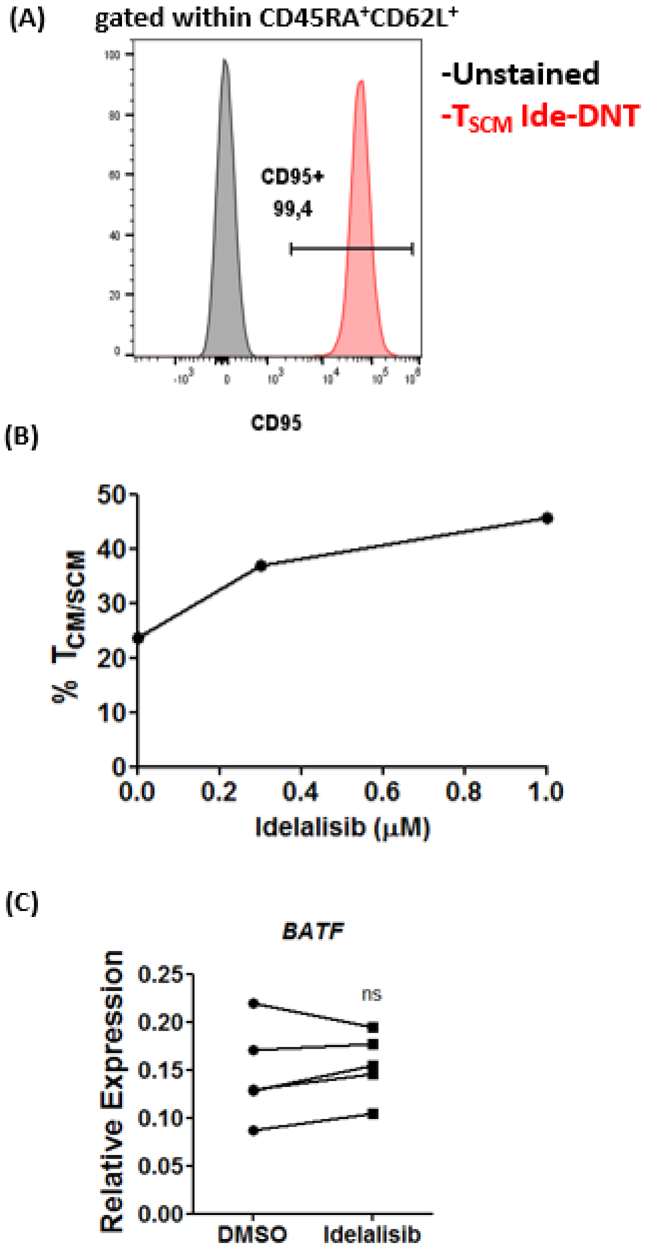

Figure S6. Ide promotes early memory subset of DNTs. Representative flow plots showing T_{SCM} (CD45RA$^+$CD62L$^+$) cells expressing CD95 using day 14 ex vivo expanded DNTs (**A**). Frequency of DNTs in early memory subset $T_{CM/SCM}$ (CD45RA$^+$CD62L$^+$ and CD45RA$^-$CD62L$^+$) on day 17 of expanded DNTs in 0, 0.3 and 1uM of Ide (**B**). Expression of transcription factor gene important for effector T cell differentiation, *BATF*, relative to the expression of housekeeping gene, *HPRT*, determined by qPCR. Each paired symbols represents DNTs from one individual (**C**).

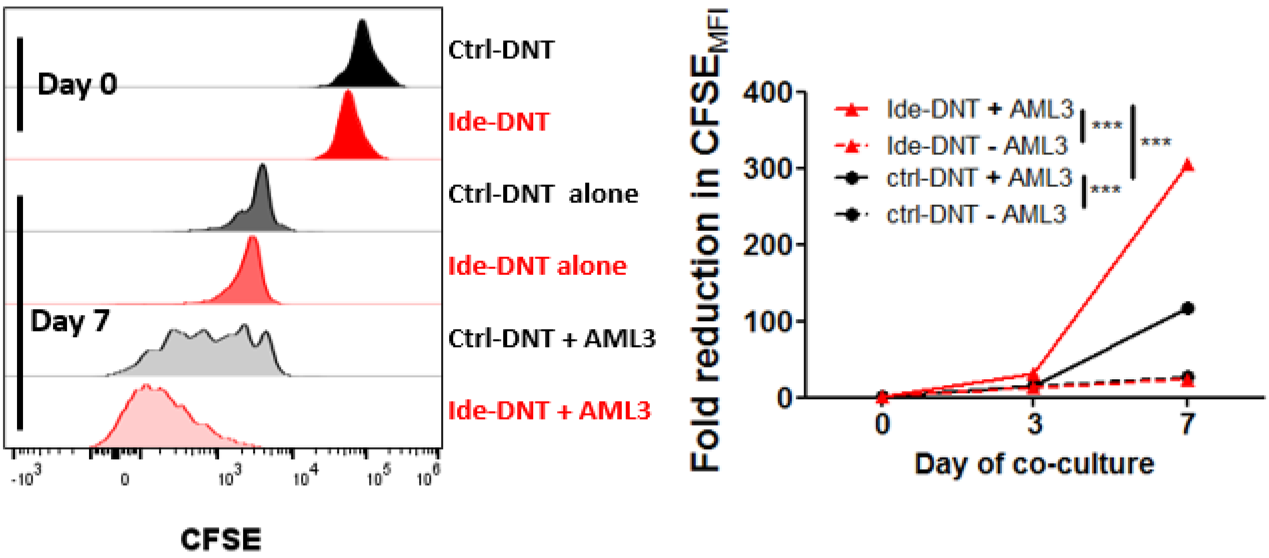

Figure S7. Ide enhances proliferative capacity of DNT after encountering AML in vitro. Expansion of ctrl-DNT (black) and Ide-DNTs (red) in the presence of DNT-susceptible AML cell line, OCI-AML3. Flow plot shows CFSE changes in DNT population ($CD3^+CD33^-$) on day 0 and day 7 after co-culture (left). Fold reduction in MFI value of CFSE was determined on 0, 3 days and 7 days post co-culture from each treatment group (right).

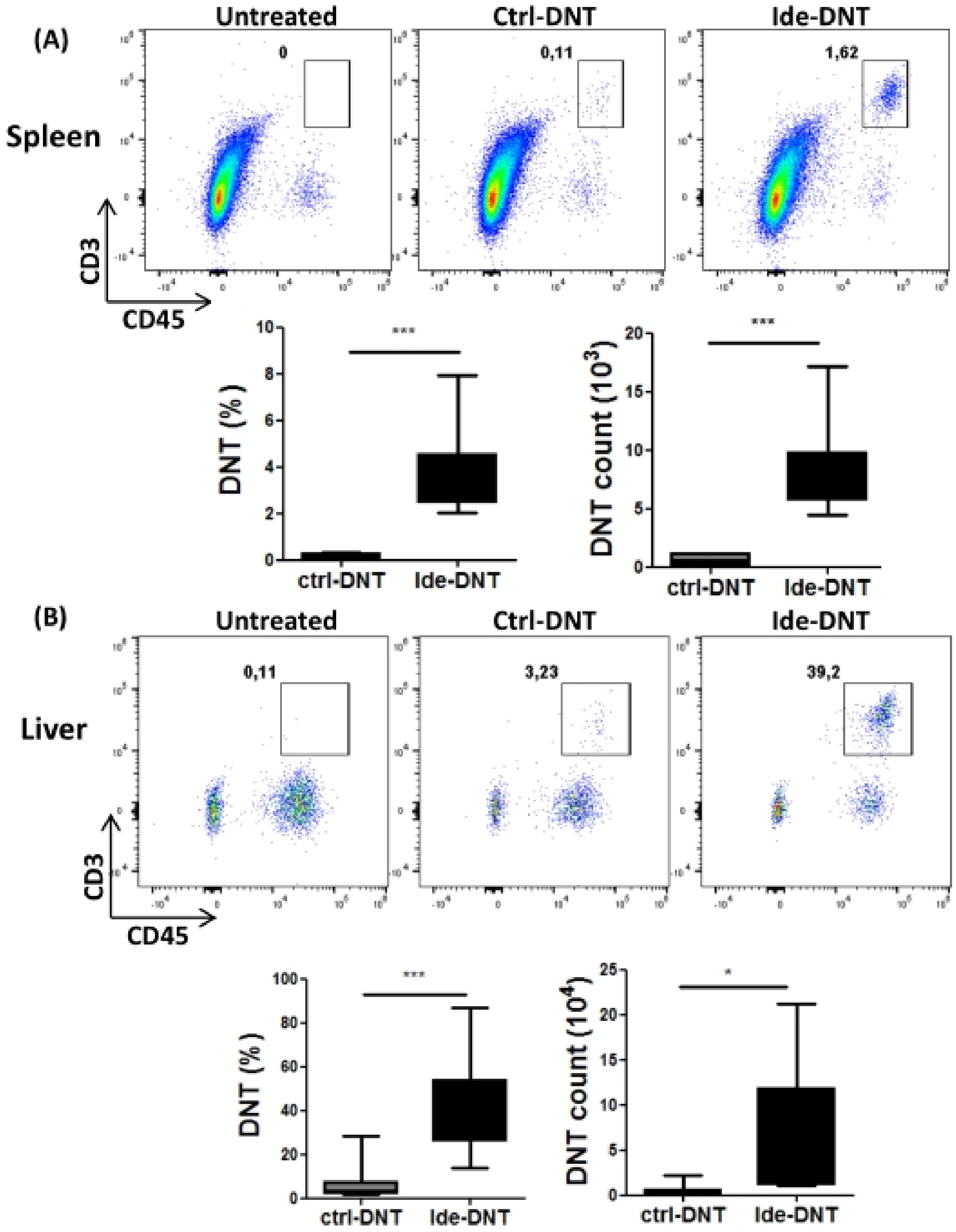

Figure S8. Idelalisib prolongs persistence of DNTs in vivo. (**A**,**B**) NSG mice engrafted with primary AML sample (090543) were treated with PBS (n = 8), ctrl-DNT (n = 8), or Ide-DNT (n = 9) on day 17 and 20 post AML infusion. Subsequently, Spleen (**A**) and liver (**B**) engraftment of DNT ($CD45^{high}CD3^{+}$) were determined on day34, 14 days post DNT infusion using flow cytometry. Representative flow plot shows DNT engraftment ($CD45^{high}CD3^{+}$).

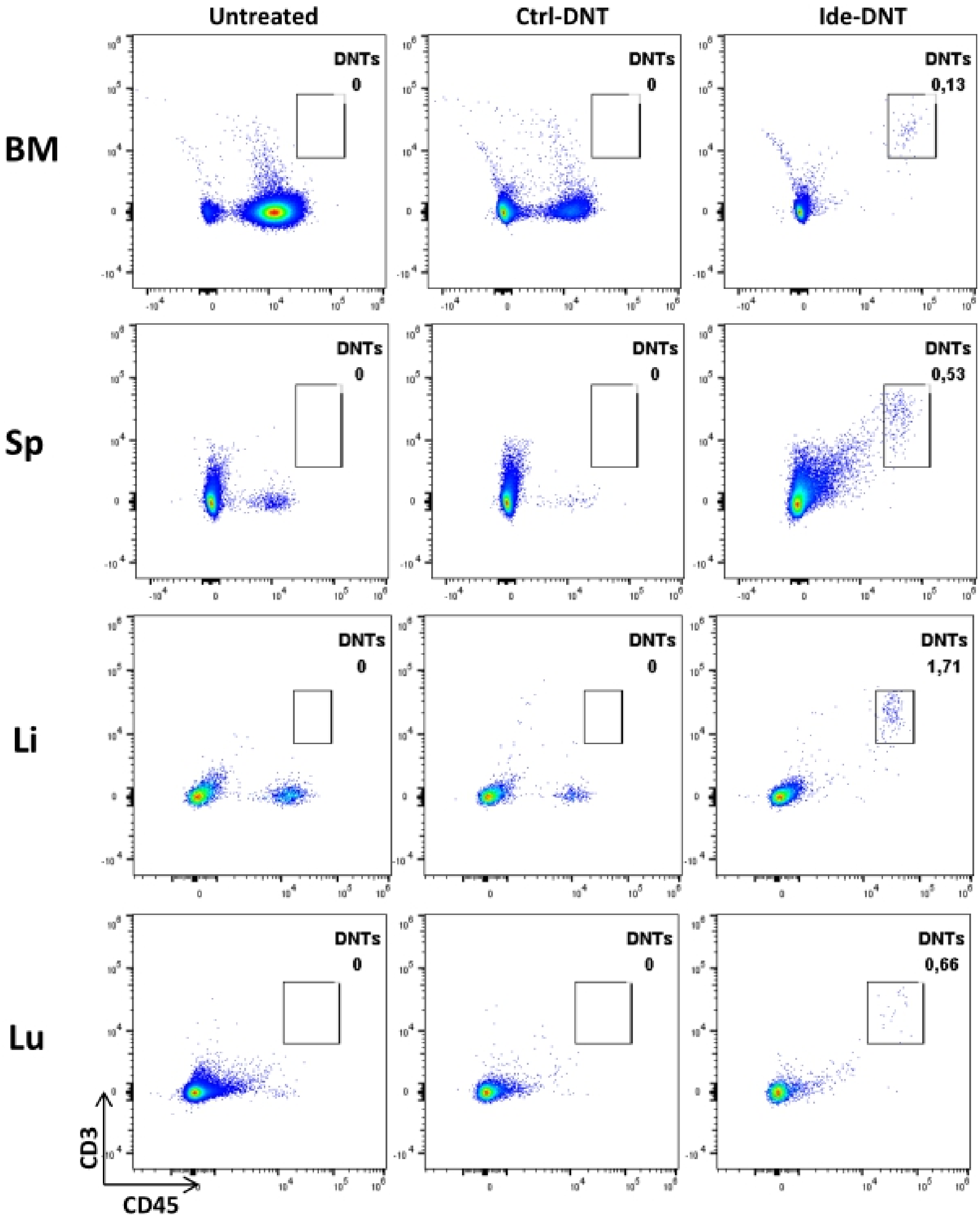

Figure S9. Ide prolongs the persistence of DNT. NSG mice engrafted with primary AML sample (090517) were Table 5. ctrl-DNT (n = 7), or Ide-DNT (n = 6), on day 17 and 20 post AML infusion. Representative flow plots show DNT engraftment (CD45^{high}CD3^{+}) from BM, spleen, liver and lung that were determined on day 100, which was 80 days post DNT infusion using flow cytometry.

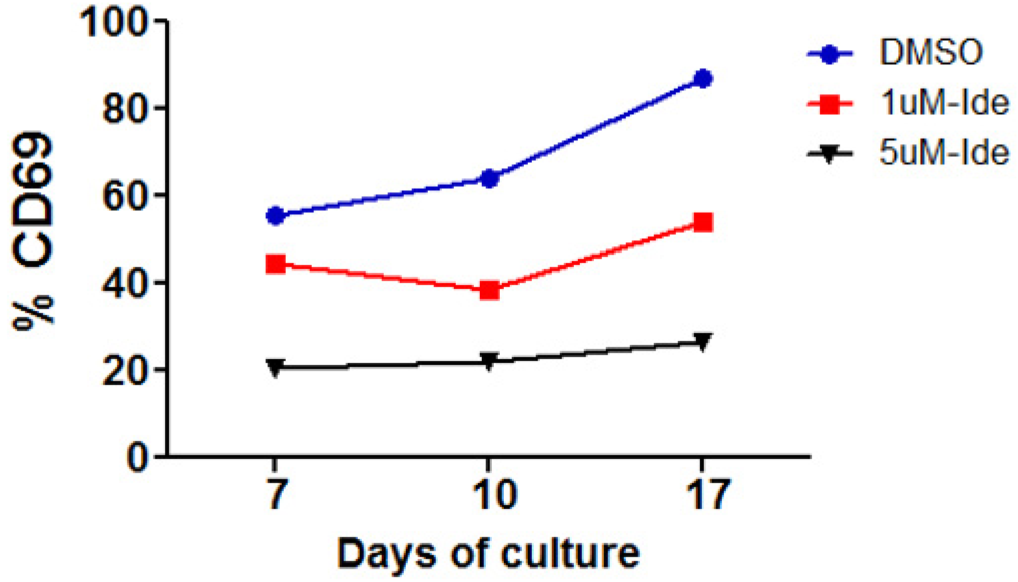

Figure S10. Idelalisib inhibits activation of DNTs. Frequency of CD69 expression from day 7, 10 and 17 ex vivo expanded DNTs in 0 (blue), 1 (red) and 5 μM (black) of Ide.

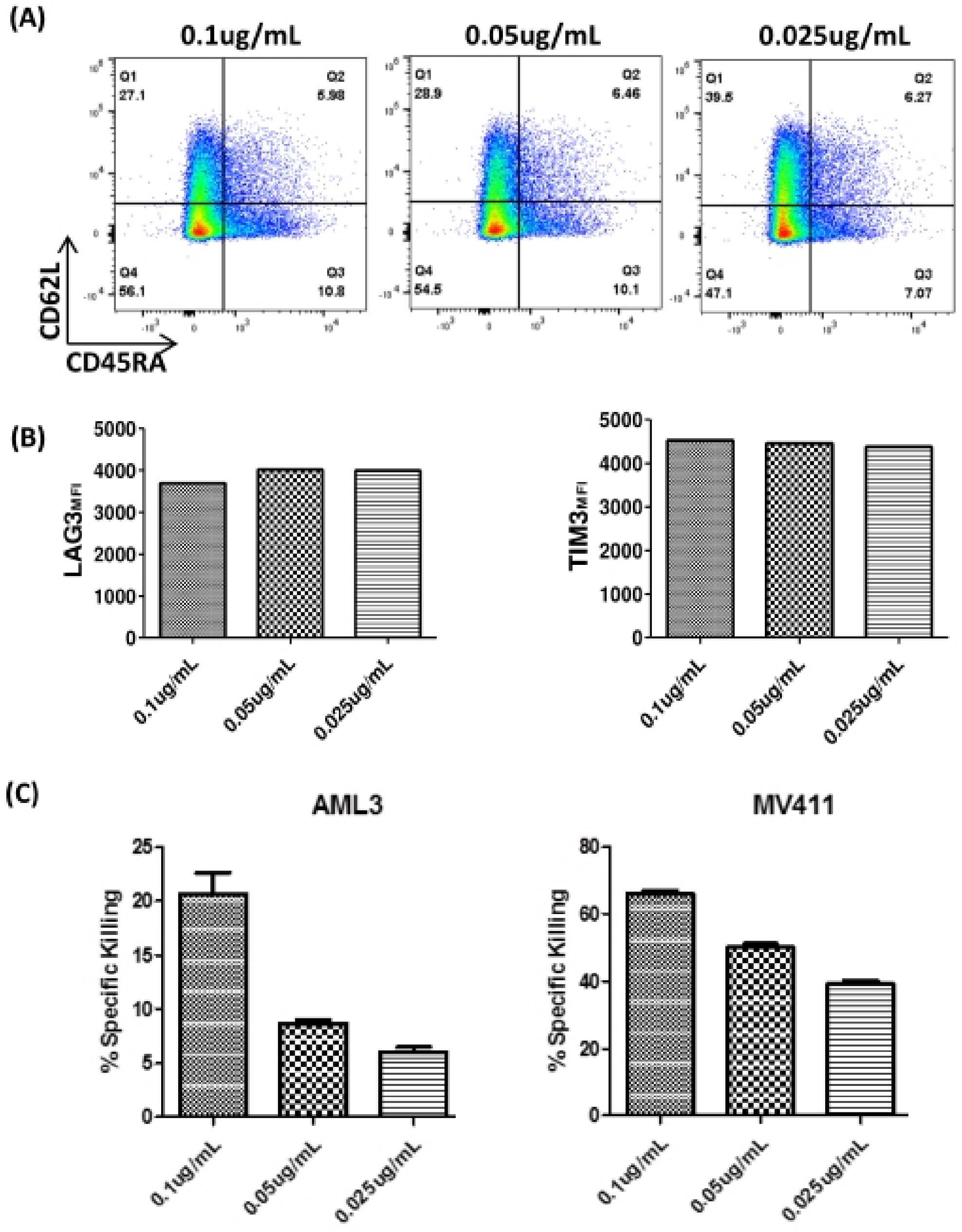

Figure S11. Lowering soluble OKT3 concentration did not affect differentiation but cytotoxicity. (**A**–**C**) DNTs expanded in 0.1, 0.05 and 0.025ug/mL of soluble OKT3 from day7 to day17. Representative flow plots showing memory status of DNTs on day 17 of expansion (**A**). T_{SCM} = $CD45RA^{+}$ $CD62L^{+}$; T_{CM} = $CD45RA^{-}$ $CD62L^{+}$; T_{EM} = $CD45RA^{-}$ $CD62L^{-}$; $T_{effector}$ = $CD45RA^{+}$ $CD62L^{-}$. Expression of exhaustion markers, LAG3 and TIM3 MFI value of DNTs on day 17 of expansion (**B**). Cytotoxicity Assay with DNTs as effector cells and AML cell lines, OCI-AML3 and MV4-11 as target cells (**C**). Effector cells were co-cultured with target cells with 0.25 to 1, effector to target ratio, overnight. Subsequently, cells were stained with 7AAD, AnnexinV, CD3, CD33. Viability of targets (gated on $CD3^{-}$ $CD33^{+}$) were determined using flow cytometry to calculate % specific killing by DNTs.

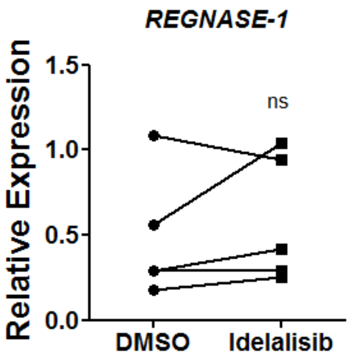

Figure S12. Idelalisib did not change the expression of REGNASE-1. Expression of *REGNASE-1* relative to the expression of housekeeping gene, *HPRT,* was determined by qPCR. Each paired symbols represents DNTs from one individual.
